# The academic, economic and societal impacts of Open Access: an evidence-based review

**DOI:** 10.12688/f1000research.8460.3

**Published:** 2016-09-21

**Authors:** Jonathan P. Tennant, François Waldner, Damien C. Jacques, Paola Masuzzo, Lauren B. Collister, Chris. H. J. Hartgerink

**Affiliations:** 1 Department of Earth Science and Engineering, Imperial College London, London, UK; 2 Earth and Life Institute, Université catholique de Louvain, Louvain-la-Neuve, Belgium; 3 Medical Biotechnology Center, VIB, Ghent, Belgium; 4 Department of Biochemistry, Ghent University, Ghent, Belgium; 5 University Library System, University of Pittsburgh, Pittsburgh, PA, USA; 6 Department of Methodology and Statistics, Tilburg University, Tilburg, Netherlands

**Keywords:** Open Citation Advantage, Altmetrics, Open Access, Publishing, Copyright, Incentive System, Open Science, Text and Data Mining

## Abstract

Ongoing debates surrounding Open Access to the scholarly literature are multifaceted and complicated by disparate and often polarised viewpoints from engaged stakeholders. At the current stage, Open Access has become such a global issue that it is critical for all involved in scholarly publishing, including policymakers, publishers, research funders, governments, learned societies, librarians, and academic communities, to be well-informed on the history, benefits, and pitfalls of Open Access. In spite of this, there is a general lack of consensus regarding the potential pros and cons of Open Access at multiple levels. This review aims to be a resource for current knowledge on the impacts of Open Access by synthesizing important research in three major areas: academic, economic and societal. While there is clearly much scope for additional research, several key trends are identified, including a broad citation advantage for researchers who publish openly, as well as additional benefits to the non-academic dissemination of their work. The economic impact of Open Access is less well-understood, although it is clear that access to the research literature is key for innovative enterprises, and a range of governmental and non-governmental services. Furthermore, Open Access has the potential to save both publishers and research funders considerable amounts of financial resources, and can provide some economic benefits to traditionally subscription-based journals. The societal impact of Open Access is strong, in particular for advancing citizen science initiatives, and leveling the playing field for researchers in developing countries. Open Access supersedes all potential alternative modes of access to the scholarly literature through enabling unrestricted re-use, and long-term stability independent of financial constraints of traditional publishers that impede knowledge sharing. However, Open Access has the potential to become unsustainable for research communities if high-cost options are allowed to continue to prevail in a widely unregulated scholarly publishing market. Open Access remains only one of the multiple challenges that the scholarly publishing system is currently facing. Yet, it provides one foundation for increasing engagement with researchers regarding ethical standards of publishing and the broader implications of 'Open Research'.

## Introduction

Open Access (OA) refers to the removal of major obstacles to accessing, sharing and re-using the outputs of scholarly research. The rationale is that the research process is facilitated by ensuring rapid and widespread access to research findings such that all communities have the opportunity to build upon them and participate in scholarly conversations. As such, the major drivers behind OA relate to within- and between-community equality ( Veletsianos & Kimmons, 2012), as well as bridging the global North-South research divide ( Adcock & Fottrell, 2008). Reflecting this ambition, there are currently over 700 OA policies and mandates recorded worldwide from a range of research institutes and funding bodies ( roarmap.eprints.org). OA pertains to documents made available via two main pathways: the Gold route and the Green route ( Harnad *et al.*, 2008). The Gold route refers to freely accessible research articles at the point of publication. This route is often, although not always, accompanied by article processing charges (APCs). The Green route refers to author self-archiving, in which peer-reviewed articles and/or not peer-reviewed pre-prints are posted online to an institutional and/or subject repository, or to a personal website. This route is often dependent on journal or publisher policies on self-archiving ( sherpa.ac.uk/romeo). Some publishers require an embargo period before deposition in public repositories is allowed. These embargoes are applied in order to avoid putative reductions in subscription income due to such self-archiving, although there is little evidence to support the existence of such embargoes ( Berners-Lee *et al.*, 2005; Bernius *et al.*, 2013; Henneken *et al.*, 2006; Houghton & Oppenheim, 2010; Swan & Brown, 2005). The Green route is also enabled through author rights retention, in which authors pre-emptively grant non-exclusive rights to their institutions before publishing any works. The institution then has the ability to make articles by these authors OA without seeking permission from the publishers (e.g., this is the case of the Dutch Taverne amendment that has declared self-archival of research after ‘a reasonable period of time’ a legal right ( Open Access NL, 2015)). Through these dual pathways, almost 25% of all scholarly documents archived on the Web are now obtainable via OA somewhere on the Internet ( Khabsa & Giles, 2014).

A core issue remains: universal or even marginal access to approximately 75% of articles is not directly possible unless one either is in a privileged position to work at an institute that has subscription access to these articles, or has enough money to pay on a per-article basis (given that journals provide this feature; some do not). Subscriptions to all peer-reviewed journals is not affordable for any single individual, research institute or university ( Odlyzko, 2006; Suber, 2012). Consequently, the potential impact of research articles is never fully realized, impeding scientific progress by a lack of use, while simultaneously negatively affecting the recognition of individual researchers ( Hitchcock, 2013) and the funders who support their work.

Because of these issues, free and unrestricted access to primary research literature has become a global goal of the OA movement. The steady increase in OA over the past two decades has required careful negotiations between a range of stakeholders (e.g., librarians, funders, academics). Much of the driving force behind this global change has been through a combination of direct, grassroots advocacy initiatives and policy reforms from universities, funders and governments. The debates regarding the benefits of OA over subscription-based access often hinge on the increased value to academics. However, increased access has broader benefits to research through enhanced visibility, facilitating innovation by businesses and decreasing financial pressure on academic/research libraries (known more broadly as the ‘serials crisis’ ( McGuigan & Russel, 2008)). Additionally, increased access to scholarly outputs might help foster a culture of greater scientific education and literacy, which in turn could have a direct impact on public policy ( European Commission, 2012; Zuccala, 2010), particularly in domains such as climate change and global health, as well as increasing public engagement in scientific research ( Stodden, 2010). OA also includes a moral aspect, where access to scientific knowledge and information is regarded as a fundamental feature of global human equality. For example, Article 27 of the United Nations Declaration of Human Rights states that " *Everyone has the right to freely participate in the cultural life of the community, to enjoy the arts and to share in scientific advancement and its benefits.*" ( United Nations, 1948).

This review aims to provide information on the various impacts of OA to scholarly research. We consider the impact of OA from the academic, economic, and societal perspective. In addition, we shortly consider the broader implications of OA on Open Data, a closely related aspect united under a general theme of Open Research or Open Science. By aggregating evidence from a range of primary sources, this review should be useful to those broadly interested in the impact of open scholarly research, as well as policymakers and others involved in implementing OA policies and strategies. We refrain from making predictions about the future of OA publishing or policy recommendations, as these are both beyond the scope of this work.

## A brief history of Open Access

The OA movement is intrinsically tied to the development of the Internet and how it redefined communication and publishing ( Laakso *et al.*, 2011). With increased availability of Internet bandwidth, print articles have become virtually redundant, and sharing of information has never been cheaper. As a consequence, the costs per research article should have potentially decreased as a result of not investing material resources in publications printing and distribution. Therefore, widespread dissatisfaction with the expensive traditional publishing model has increased, resulting in the OA movement and concomitant innovations in scholarly publishing. A comprehensive timeline of the OA movement is provided as part of the Open Access Directory ( oad.simmons.edu/oadwiki/Timeline).

Interest in using the Internet for facilitating access to scientific research coalesced throughout the 1990s, culminating with the 2001 conference on "Free Online Scholarship" by the Open Society Institute in Budapest. The result of this conference was the release of the Budapest Open Access Initiative (BOAI), which is recognized as one of the defining points of the OA movement. The BOAI was the first initiative to use the term "Open Access" and articulated the following definition:

*By "open access" to [peer-reviewed research literature], we mean its free availability on the public internet, permitting any users to read, download, copy, distribute, print, search, or link to the full texts of these articles, crawl them for indexing, pass them as data to software, or use them for any other lawful purpose, without financial, legal, or technical barriers other than those inseparable from gaining access to the internet itself. The only constraint on reproduction and distribution, and the only role for copyright in this domain, should be to give authors control over the integrity of their work and the right to be properly acknowledged and cited.*

This definition is broadly equivalent to the Creative Commons Attribution license (CC-BY), which is widely considered to be a standard for OA ( creativecommons.org/licenses/). One result of the growing OA movement is the rise of OA-only publishers, who publish exclusively digital content and have demonstrated that such a business model is financially feasible (but does not necessarily sustain the current journal ecosystem). Some of these publishers are for-profit and some are non-profit. For example, pioneer OA publishers BioMed Central (for-profit) and the Public Library of Science (PLOS) (non-profit) were founded in the early 2000s and remain successful OA publishing businesses to date. More recently, OA publishing has gained increasing momentum among researchers, funders, and governments. This has led to a proliferation of innovative approaches to publishing (e.g., *PeerJ*, peerj.com; *F1000Research*, f1000research.com; *Open Library of Humanities*, openlibhums.org) and a range of different policies from research funders and institutes mandating OA. All of these different policies and new business models, combined with traditional publishers launching their own OA titles and programs, have made the overall OA ecosystem quite complex.

**Figure 1.**
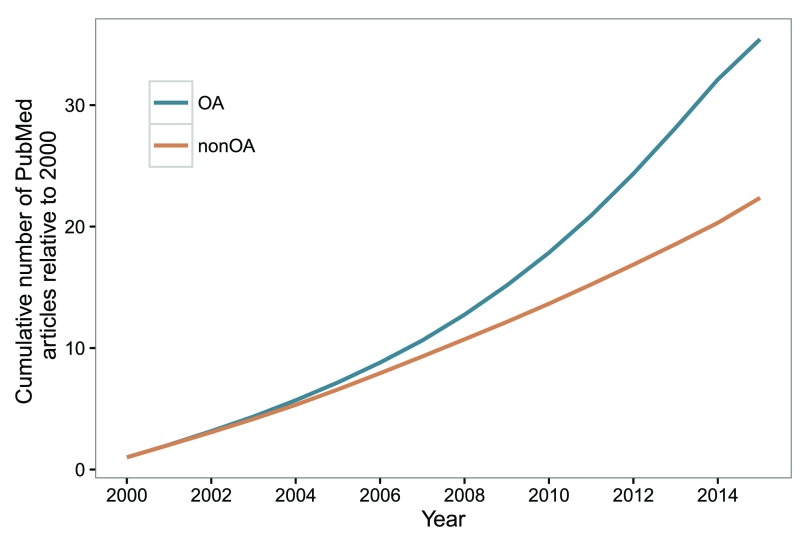
Percentage increase in research articles in PubMed Central, relative to 2000. Since 2004, the growth rate of OA articles is significantly higher than that of non-OA articles.

Even with this growing prevalence of publishers that facilitate OA to the scholarly literature, OA is still hardly ubiquitous. Bjork *et al.* (2009) estimated that the total number of published articles in 2006 was approximately 1,350,000. Of these, 4.6% became immediately accessible and an additional 3.5% became accessible after an embargo period of typically one year. Furthermore, usable copies of 11.3% could be found in repositories or on the author’s home pages. Since the U.S. National Institutes of Health (NIH) mandated archival of articles in the public PubMed Central repository in 2008, the cumulative number of OA articles in PMC has increased more than the number of non-OA articles (see Figure 1). In 2013, the total percentage of OA articles available was estimated at 24% of English-language scholarly documents accessible on the Web ( Khabsa & Giles, 2014).

Although these estimates show OA is on the rise, the full potential of OA is far from achieved. Björk *et al.* (2014) pointed out that 62% of journals (from the top 100 journal publishers indexed by SCOPUS) endorse immediate Green OA self-archiving by their authors, 4% impose a 6-month embargo, and 13% impose a 12-month embargo. As such, 79% of articles published in any recent year could already be OA within 12 months after publication via Green OA, 62% of them immediately if authors were actually self-archiving properly ( Gargouri *et al.*, 2010; Gargouri *et al.*, 2012). The disconnect between practice and what is allowed has three potential explanations: (i) researchers are unsure whether they have the legal right to self-archive, (ii) they fear that it might put their article’s acceptance for publication at risk, and (iii) they believe that self-archiving may be a lot of work ( Harnad, 2006). Research funders and institutions worldwide are now beginning to realize that they need to alter their conditions to make OA *mandatory* ( Vincent-Lamarre *et al.*, 2016) in order to counteract these misconceptions of self-archiving ( Carr *et al.*, 2007; Swan & Brown, 2005). Swan & Brown (2005) have indicated that the vast majority of researchers (81%) would comply with mandatory OA if it were a condition of funding. On the other hand, it is worth mentioning that ensuring compliance with OA policies set by research institutions is rather difficult. Some tools, such as the *Open Access Monitor* ( http://symplectic.co.uk/elements-updates/introducing-open-access-monitor), help institutions to track compliance with their OA policy.

Table 1 shows a non-exhaustive summary of the developments in the advancement of scholarly publishing and the OA movement. Included are the founding of major institutions in the movement as well as policy and legal developments. Several controversial moments are included, because they have spurred action or generated awareness for the movement. One of them is the suicide of Aaron Swartz, who was arrested for downloading JSTOR articles on the grounds that he allegedly intended to make these publicly available. Another ongoing controversy is scholarly piracy; this includes the Sci-Hub and LibGen projects, which have created an online repository of pirated scholarly papers (around 50 million at the time of writing). Both projects gained increased attention after becoming the target of a lawsuit by the publisher Elsevier. There have been mixed responses to these kinds of activities, polarising the view that illegal acts regress or weaken the case for OA, while some hail the development as the ‘Napster moment’ (i.e., a change inducing disruption; Rosenwald, 2016) for the OA movement, which will force the established industry to change. Regardless of the legality of it, Sci-Hub is used by a large number of people from all over the world to access research articles ( Bohannon, 2016; Elbakyan & Bohannon, 2016).

**Table 1.** Major historical milestones in the progress of Open Access publishing.

Y ear	M ilestone
1454	Invention of **printing**
1665	January 5: First issue of The ***Journal des sçavans*** (later spelled *Journal des savants*), the earliest academic journal published in Europe and established by Denis de Sallo.
1807	25-year-old **Charles Wiley** opens a small printing shop at 6 Reade Street in lower Manhattan.
1842	May 10: **Julius Springer** founded what is now Springer Science+Business Media in Berlin.
1848	**John Wiley** (son of Charles Wiley) gradually started shifting his focus away from literature toward scientific, technical, medical, and other types of nonfiction publishing.
1880	Foundation of **Elsevier**.
1936	First scientific book published by **Elsevier**.
1990	First **web page**.
1991	An online repository of electronic preprints, known as e-prints, of scientific papers is founded in Los Alamos by the American physicist Paul Ginsparg. It was renamed to **ArXiv.org** in 1999. The total number of submissions by May 11st, 2016 (after 24.8 years) is 1,143,129 ( arxiv.org/stats/monthly_submissions).
1993	Creation of the **Open Society Institute** (renamed to the Open Society Foundations [OSF] since 2001) by the progressive liberal business magnate George Soros. The OSF financially supports civil society groups around the world, with a stated aim of advancing justice, education, public health and independent media.
1997	Launch of **SciELO** in Brazil. There are currently 14 countries in the SciELO network and its journal collections: Argentina, Bolivia, Brazil, Chile, Colombia, Costa Rica, Cuba, Mexico, Peru, Portugal, South Africa, Spain, Uruguay, and Venezuela.
1998	**Public Knowledge Project** (PKP) is founded by John Willinsky in the Faculty of Education at UBC, with Pacific Press Professorship endowment, dedicated to improving the scholarly and public quality of research.
	PKP has created the **Open Conference Systems** (2000), **Open Journal Systems** (2001), **Open Harvester Systems** (2002) and the **Open Monograph Press** (2013).
2000	**BioMed Central**, the self-described first and largest OA science publisher and **PubMed Central**, a free digital repository for biomedical and life sciences journal, is founded. In 2008, Springer announces the acquisition of BioMed Central, making it, in effect, the world’s largest open access publisher.
2001	An online petition calling for all scientists to pledge that from September 2001 they would discontinue submission of papers to journals which did not make the full-text of their papers available to all, free and unfettered, either immediately or after a delay of several months is released. The petition collected 34,000 signatures but publishers took no strong response to the demands. Shortly thereafter, the **Public Library of Science** (PLOS) was founded as an alternative to traditional publishing. *PLOS ONE* is currently the world’s largest journal by number of papers published (about 30,000 a year in 2015).
	December 1–2: **Conference convened in Budapest** by the Open Society Institute to promote open access – at the time also known as Free Online Scholarship. Where the Budapest Open Access Initiative (BOAI) was born.
2002	February 14th: Release of the **Budapest Open Access Initiative** (BOAI), a public statement of principles relating to OA to the research literature. This small gathering of individuals is recognised as one of the major defining events of the OA movement. On the occasion of the 10th anniversary of the initiative, it was reaffirmed in 2012 and supplemented with a set of concrete recommendations for achieving "the new goal that within the next ten years, Open Access will become the default method for distributing new peer-reviewed research in every field and country."
	Start of the Research in Health - **HINARI** programme of the World Health Organization and major publishers to enable developing countries to access collections of biomedical and health literature online at reduced subscription costs. Together with Research in Agriculture - **AGORA**, Research in the Environment - **OARE** and Research for Development and Innovation - **ARDI** programmes, it currently forms **Research4Life** that provides developing countries with free or low cost access to academic and professional peer-reviewed content online.
2008	The **National Institutes of Health** (NIH) Public Access Policy, an OA mandate requiring that research papers resulting from NIH funding must be freely and publicly available through PubMed Central within 12 months of publication, is officially recorded.
2009	The **Fair Copyright in Research Works Act** (Bill H.R 801 IH, also known as the "Conyers Bill") is submitted as a direct response to the National Institutes of Health (NIH) Public Access Policy; intending to reverse it. The bill’s alternate name relates it to U.S Representative John Conyers (D-MI), who introduced it at the 111th United States Congress on February 3, 2009.
2011	Arrest of **Aaron Swartz** after he systematically downloaded articles from JSTOR, for alleged copyright infringement.
	In reaction to the high cost of research papers behind paywalls, **Sci-Hub**, the first known website to provide automatic and free, but illegal, access to paywalled academic papers on a massive scale, is founded by Alexandra Elbakyan from Kazakhstan.
2012	Start of the **Academic Spring**, a trend wherein academics and researchers began to oppose restrictive copyright in traditional academic journals and to promote free online access to scholarly articles.
	Start of the **Cost of Knowledge** campaign which specifically targeted Elsevier. It was initiated by a group of prominent mathematicians who each made a commitment to not participate in publishing in Elsevier’s journals, and currently has over 15,933 co-signatories.
	Start of the United States-based campaign **Access2Research** in which open access advocates (Michael W. Carroll, Heather Joseph, Mike Rossner, and John Wilbanks) appealed to the United States government to require that taxpayer-funded research be made available to the public under open licensing. This campaign was widely successful, and the directive and FASTR (the Fair Access to Science and Technology Research Act) have become defining pieces in the progress of OA in the USA at the federal level.
	Launch of ***PeerJ***, an OA journal that charges publication fees through researcher memberships, not on a per-article basis, resulting in what has been called "a flat fee for ’all you can publish’". Note that as of October 2015 *PeerJ* also have a flat rate APC of $695.
2013	January: The suicide of **Aaron Swartz** draws new international attention for the Open Access movement.
	November: **Berlin 11 Satellite Conference** for students and early career researchers, which brought together more than 70 participants from 35 countries to engage on Open Access to scientific and scholarly research.
2014	First **OpenCon** in Washington DC, an annual conference for students and early career researchers on Open Access, Open Data, and Open Educational resources.
	Open Access is embedded the European Commission’s **Horizon 2020** Research and Innovation programme.
2015	Academic publisher **Elsevier** makes a complaint in New York City for copyright infringement by **Sci-Hub**. Sci-Hub is found guilty and ordered to shut down. The website re-emerges under a different domain name as a consequence. A second hearing in March 2016 is delayed due to failure of the defendant to appear in court, and to gather more evidence for the prosecution.

## The effect of Open Access upon academia

The two main ways in which OA affects academia are (i) through association with a higher documented impact of scholarly articles, as a result of availability and re-use, and (ii) through the possibility of non-restrictively allowing researchers to use automated tools to mine the scholarly literature. For the former, major arguments in favor of OA include the evidence that work that is openly available generates more academic citations, but also has more societal impact. In addition, appropriately-licensed OA works play a major role in academic education, including re-use in classes and for dissertations. The latter major argument involves non-restrictive access to the scholarly literature through appropriate licensing, making it possible to use automated tools to collect and analyze the entire body of scholarly literature in a legally sound framework and irrespective of copyright laws. The following sections cover these two effects of OA.

### The potential impact advantage

**Figure 2.**
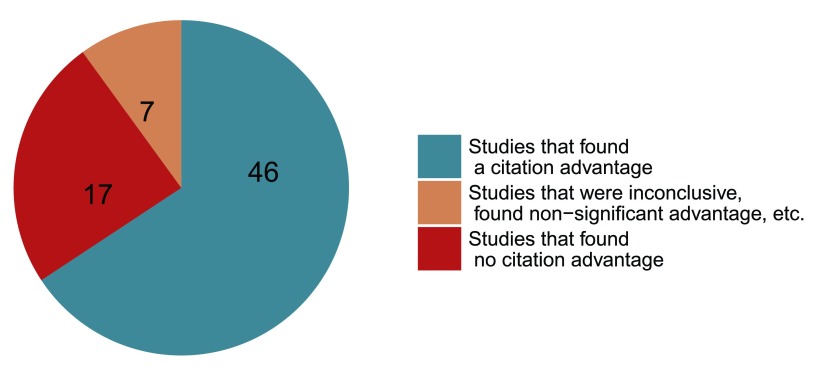
Studies that investigated the citation advantage grouped by their conclusion. The majority concluded that there is a significant citation advantage for Open Access articles. Source: Data from The Open Access Citation Advantage Service, SPARC Europe, accessed March 2016.

***Academic impact.*** Academic impact is frequently measured through citation counts, and these remain fundamental as the ‘currency units’ for researchers, research groups, institutes and universities. Lawrence (2001) was the first to propose that OA would have a citation advantage. The utility and consistency of the citation advantage across different research fields has been intensively debated because its magnitude substantially varies depending on the discipline ( Table 2). However, the general tendency identified by studies to date indicates that there is at least some association between OA publishing and increased citation counts across most disciplines ( Hajjem *et al.*, 2006; Antelman, 2004) ( Figure 2 and Table 2). A comprehensive and annotated bibliography of studies documenting potential citation impacts was created by Steve Hitchcock ( eprints.soton.ac.uk/354006/1/oacitation-biblio-snapshot0613.html) and has been managed by SPARC Europe since 2013 ( sparceurope.org/oaca/).

**Table 2.** Main scientific papers that have investigated and quantified the citation advantage as well as its origin.

R eference	D iscipline	C itation advantage	O rigin
Antelman (2004)	Mathematics, Electrical Engineering, Political Science, Philosophy	+91%, +51%, +86%, +45% per discipline respectively	NA
Atchison & Bull (2015)	Political Science	Statistically significant citation advantage	NA
Cheng & Ren (2008)	Medicine, Biology, Agricultural Sciences, Chemistry and University Journals	+200%	NA
Davis & Fromerth (2007)	Mathematics	+35%	Quality advantage, no evidence of early advantage
Davis *et al.* (2008)	Physiology	-5%	NA
Davis (2011)	Sciences, Social Sciences, and Humanities	+1% but statistically indistinguishable	No evidence of an early advantage
Evans & Reimer (2009)	All	+8% for newly published articles; +16% for citations from developing countries	NA
Eysenbach (2006)	Natural Sciences	+210 up to +290%	NA
Frandsen (2009)	Biology, Mathematics, Pharmacy and Pharmacology	No clear tendency towards an increase in impact	NA
Gargouri *et al.* (2010)	Engineering, Biology, Biomedicine, Chemistry, Psychology, Mathematics, Clinical Medicine, Health, Physics, Social Science, Earth Sciences	+?% to ?% depending on the discipline	Quality advantage is confirmed no evidence for selection bias
Gaule & Maystre (2011)	Biology	No evidence of citation advantage	NA
Gentil-Beccot *et al.* (2010)	High Energy Physics	+200%	Early advantage confirmed
Hajjem *et al.* (2006)	Biology, Psychology, Sociology, Health, Political Science, Economics, Education, Law, Business, Management	+36% to 172%	NA
Harnad & Brody (2004)	Physics	+250% to 580%	NA
Henneken *et al.* (2006)	Astronomy and Physics	+200%	NA
Kousha & Abdoli (2010)	Agricultural Science	+621% but not to every journal	NA
Kurtz *et al.* (2005)	Astronomy	None	Selection bias and early advantage
Kurtz & Henneken (2007)	Astronomy	+200%	Early advantage confirmed
Lansingh & Carter (2009)	Opthalmology	No	NA
Lawrence (2001)	Computer Science	+157% up to +284% for top publication	NA
McCabe & Snyder (2014)	Ecology, Botany, Multidisciplinary Science and Biology	+8%	NA
McVeigh (2004)	Natural Sciences	0-+50% in 2003 depending on field, negative citation advantage in 2000	NA
Metcalfe (2005)	Astronomy	+200%	NA
Metcalfe (2006)	Solar Physics	+170% and +260% depending on the online repository	No evidence for selection bias
Moed (2006)	Condensed Matter Physics	NA	Confirm early access advantage and selection bias but no OA effect
Norris *et al.* (2008)	Ecology, Applied Mathematics, Sociology and Economics	+157%	NA
Sahu *et al.* (2005)	Medicine	+300% up to +450%	NA
Schwarz & Kennicutt Jr (2004)	Astronomy	+200%	Early advantage
Vanclay (2013)	Environmental Science	Not significant	NA
Wang *et al.* (2015)	All	+111% up to 152%	NA
Wohlrabe & Birkmeier (2014)	Economics	+35% up to 64% depending on the database used	NA
Xu *et al.* (2011)	Humanities, Life Sciences, Mathematics & Physical Science, Medicine, Social Sciences	-49.24%-+87.73%	NA
Zhang (2006)	Communication Studies	+200%	NA

Estimates for the open citation advantage range from +36% (Biology) to +600% (Agricultural Sciences) ( Swan, 2010; Wagner, 2010). In a longitudinal study, Eysenbach (2006) compared the bibliometric impact of a cohort of articles from a multi-disciplinary journal ( *Proceedings of the National Academy of Sciences*) that offers both OA and non-OA publishing options. After adjusting for potentially confounding variables, the results indicated that non-OA papers were twice as likely to remain uncited six months after publication when compared to OA articles. Additionally, the average number of citations for OA articles was more than double than that of the non-OA articles. The study also differentiated the type of OA article, namely the self-archived (i.e., Green OA) and the publisher version of record (VOR) that is freely available (i.e., Gold OA). Gold OA was found to have a higher overall academic impact than Green OA.

Despite strong evidence for a citation advantage, the magnitude of this advantage remains variable. The substantial heterogeneity in observed citation advantages can be due to different academic cultures or could simply be spurious. For example, self-archiving prior to publication is a community standard in fields such as high energy physics or mathematics, but has yet to be widely adopted among the life sciences. Such ‘pre-prints’ have also been associated with an overall increase in the average number of citations, the total number of citations, and the speed of citation accumulation ( Aman, 2014; Gentil-Beccot *et al.*, 2010). Other studies could only replicate immense citation advantages (+600%) if relevant predictors were omitted ( McCabe & Snyder, 2014), which indicates a potential spurious effect. When taking into account these relevant predictors, the citation advantage became much smaller (+8%). When the citation advantage is low or non-existent, this could suggest that in those research fields there is a sufficient level of access to the literature such that OA confers no localised access advantage, or that adoption of OA has not yet reached a level where any such advantage has become statistically evident.

One alternative explanation for the existence of citation advantages could be that researchers choose to publish OA when a finding is more impactful, but empirical evidence contradicts this selection effect. Gargouri *et al.* (2010) compared citation counts for articles which were self-selected as OA or mandated as OA (e.g., by funders). The study concluded that both were cited significantly more than non-OA articles and showed no differences in citation rates. As such, these findings rule out a selection bias from authors as the cause for the citation advantage ( Gargouri *et al.*, 2010). However, research that is selected to merit funding by funding agencies may, in itself, be perceived to be more impactful than research that is not funded. Additionally, as no single OA mandate is ever 100% effective, it might be the simple case that authors are more likely to comply with a mandate for the research they perceive to be of higher impact. In a study of articles in the field of psychology, Anderson (2013a) found that publications with funding sources reported in the text were found to be more highly cited and connected to other highly-cited publications (this type of publication is called "generative" in the study) than publications with no reported funding sources. Furthermore, research that was privately funded was found to be more generative than publicly funded research. In a similar study in the Library and Information Sciences field done by Zhao (2010), the citation counts for grant-funded publications were "substantially higher" than publications without grant funding. Although these studies indicate that grant funding is correlated with increased citation rates, the openness of articles was not addressed in either study. Future research will be required to demarcate the potential causality and to determine the conditions under which we could see whether or not OA has an effect on citation counts. For example, this could be conducted through a randomised controlled trial in which research articles from a particular funder are randomly assigned to OA and non-OA routes, with the citation counts assessed after a certain time.

In sum, evidence indicates that OA is broadly related to increased academic impact in terms of citations ( Figure 2; see also McKiernan *et al.* (2016)), but given the large variability in results, further research should aim to synthesize these findings in a meta-analysis and try to explain the cause of this variability.

***Broader societal impact.*** Scholarly articles also have a societal impact, such as when they are covered in news media or are discussed in social media channels; alternative metrics, or altmetrics, can be used as a guide to measure this mode of impact ( Liang *et al.*, 2014). Information such as social media usage, Mendeley readership, and media attention ( Piwowar, 2013) can be tracked by various altmetrics providers (e.g. ImpactStory, Plum Analytics, and Altmetric.com). As such, when an article generates discussions outside of the academic literature, altmetrics are capable of tracking this. Despite limitations (such as academics discussing their own research on platforms like Twitter), altmetrics provide a general view of the wider societal impact of research articles. Considering the increased pressure on researchers and research institutes to communicate research findings to the public, altmetrics can provide additional insight into which research drives public interest. A working group established by NISO is investigating the future role of altmetrics in research communication and assessment ( www.niso.org/topics/tl/altmetrics_initiative/).

OA articles would be expected to have an altmetrics advantage compared to the non-OA literature; if an article has fewer restrictions for journalists, citizens, businesses, and policy-makers, it seems logical that this would enable the research to be publicly re-used. Furthermore, those parties may be more likely to promote articles which are publicly accessible into different communication channels. In other words, increased access removes barriers to widespread societal engagement, whereas a relative lack of article access discourages engagement.

There is research showing evidence for an altmetrics advantage for OA articles, but this does not reflect itself in the most impactful articles. Wang *et al.* (2015) found evidence that OA articles receive more attention through social media. The authors compared social media attention (Twitter and Facebook) between OA and non-OA articles at *Nature Communications* and found that OA articles get 1.2–1.48 times as much social media attention as compared to non-OA articles (see also Adie, 2014). Nonetheless, of the top 100 articles of 2015 as presented by Altmetric.com, only 42 articles were OA ( www.altmetric.com/top100/2015/). This 42% is larger than the overall proportion of OA articles in the literature, which indicates that OA contributes relatively more impact per paper. However, it also indicates that the open impact advantage can be overshadowed by the intrinsic nature of the research published or by the traditionally prestigious journals with a larger and dedicated media apparatus (e.g., Nature, Science; Brembs *et al.*, 2013).

Allen *et al.* (2013) found that a social media announcement of the release of a research article increases the number of users who view or download that article, but does not translate to increases in the citation count in the field of clinical pain research. Costas *et al.* (2015) found a relatively weak correlation between social media activity and citation counts for the articles in their sample (over 1.5 million article records), while Mohammadi *et al.* (2015) found that the number of Mendeley readers with a status of graduate student or faculty correlated with citation counts. When OA to the articles is factored into an analysis, there is a potential recursive relation between citation counts and altmetrics due to OA. Eysenbach (2011) indicated that there is a moderate correlation (0.42–0.72) between the tweets and citations of articles from an OA journal ( *Journal of Medical Internet Research*). Highly tweeted articles were eleven times more likely to be highly cited than less-tweeted articles, or vice versa (75% of highly tweeted articles were highly cited; 7% of less-tweeted articles were highly cited). However, it is difficult to assess causality in these cases: do research papers that have more academic impact make their way more frequently into societal discussions, or does online discourse increase their potential citation rates? Overall, this evidence implies that there is a general media advantage with OA (see also McKiernan *et al.* (2016)), which can be used as a proxy or pathway to indicate greater societal impact.

Altmetrics themselves should not be conflated with citations when it comes to assessing impact, even though some providers such as Altmetric.com supply a single score that can be used to rank an article in a similar way to a journal’s Impact Factor. Each measure of altmetrics tells a different story about the impact of research, and a careful understanding of the altmetrics landscape in conjunction with citation-based metrics can lead to a clearer picture of societal impact of scientific research.

### Open Access and text- and data-mining

Traditionally, in order to publish a paper, researchers hand over their copyright via a Copyright Transfer Agreement. Copyright transfer as the default has far-reaching consequences on the ability of both the authors and others to re-use that published research, and many authors are not aware of the impact of these transfers on their ownership of the work. Academics frequently give the copyright to the publishers in exchange for the perceived prestige of publishing in one of their venues (e.g., Müller-Langer & Watt, 2010). In some cases, institutes adopt rights-retention OA policies that grant authors non-exclusive rights to their institutes before signing copyright agreements with publishers, which enables them to make articles OA without requiring permission from publishers ( cyber.law.harvard.edu/hoap/Good_practices_for_university_open-access_policies). Essentially, copyright is a pre-digital tool wielded by traditional publishers to maintain revenues rather than fostering creativity, innovation, or protecting authors ( Okerson, 1991; Willinsky, 2002). For example, the Author’s Guild sued Google Books for copyright infringement because they provided freely available digital copies; the court rejected this suit in 2016, stating that Google Books served the public interest and that copyright’s "primary intended beneficiary is the public" ( EFF, 2015). In the digital age, copying is essential to perform necessary research tasks. These activities range from viewing the article (i.e., downloading requires copying) to re-using figures from an article in a book. The interaction of OA and copyright is complex and deserves extended research in itself (e.g., Scheufen, 2015). We will highlight how OA views copyright and relate this to its effects on text- and data-mining (TDM).

The majority of ‘born OA’ journals and publishers do not request or receive copyright from authors. Instead, publishers are granted non-exclusive rights to publish, and copyright is retained by authors through a Creative Commons license (typically CC-BY). Importantly, this represents a power shift from publisher-owned to author-owned rights to research. This model of author-retained copyright appears to be favoured by the majority (71%) of the research community ( Hoorn & van der Graaf, 2006). Shifting copyright to stay with the author, combined with appropriate open licensing, allows for wider re-use, including TDM, and forms the basis for a robust scholarly ecosystem.

As such, copyright in OA publications is non-restrictive and also allows machines to freely access it. In traditional publishing, human reading and computer reading are seen as two separate things which require different agreements, whereas OA publishing views them both in the same, non-restrictive manner. In other words, in order to mine OA journals, one only needs the technical skills to do so. In order to mine closed access journals, one needs to sign or negotiate access conditions, even if legitimate access to the articles has already been bought ( Bloudoff-Indelicato, 2015).

Automated extraction of information from scholarly research via TDM is a methodology that can be applied to investigate the scholarly literature at an enormous scale, creating new knowledge by combining individual findings. This has already proven to be useful for a large variety of applications (e.g., Glenisson *et al.*, 2005; Martone *et al.*, 2016; Swanson, 1987). Moreover, OA publishers facilitate TDM on a massive scale by allowing multiple options for collecting the literature needed. For example, PLOS is non-restrictive and allows users to scrape articles directly from the website or using its API. As a result, scraping tools can be used, such as rplos, an R package developed to search and download full-text scholarly papers ( Chamberlain *et al.*, 2016).

TDM is not only a knowledge-generation tool; it also allows for automated screening for errors and automated literature searches that renew scientific discovery. With TDM it becomes possible to easily compare one’s results with those of the published literature, identify convergence of evidence and enable knowledge discovery ( Natarajan *et al.*, 2006) or discover frequent tentative hypotheses that can be used for new research ( Malhotra *et al.*, 2013). It has already been used to make major advances in fields such as biomedicine ( Gonzalez *et al.*, 2016). TDM also allows for computer applications that can download all scholarly literature given certain search terms (e.g., ContentMine’s getpapers tool ; github.com/ContentMine/getpapers), simplifying and shortening the tedious literature search. TDM can also serve a screening purpose similar to plagiarism scanners, helping to detect statistical errors in the scholarly literature (e.g., Nuijten *et al.* (2015)). TDM can be used in various innovative ways and is an emerging and rapidly advancing field; non-restrictive licensing through OA certainly promotes its wider application.

Given the exponential increase in the number of scholarly publications, (semi-)automated methods to synthesize results have become increasingly important. TDM decreases the time dedicated to the search for relevant information in scholarly literature by categorizing information ( Leitner & Valencia, 2008), highlighting and annotating relevant results to specific users ( Shatkay *et al.*, 2008), and profiling research ( Porter *et al.*, 2002). Furthermore, TDM also prevents researchers and readers from wasting time on reinventing the wheel, simply because one can no longer keep up with the huge amount of published literature available ( Harmston *et al.*, 2010).

Because of traditional copyright transfers, TDM has often been stymied by traditional, closed access publishers who frequently see it as a copyright infringement. Researchers using software that harvests data from online publications have been (threatened to be) cut off from accessing the articles. These researchers found themselves trapped in negotiations to resume their research (even though their universities had paid subscription fees for access (e.g., Bloudoff-Indelicato, 2015; Van Noorden, 2012)). Standard subscriptions do not permit systematic downloads because publishers fear that their content might be stolen and their revenue therefore lost ( Van Noorden, 2012). In 2014, Elsevier opened its papers for TDM via a proprietary API ( Van Noorden, 2014), but placed restrictions on the researchers using it; however, researchers are not legally required to comply with these restrictions in some countries (e.g., U.K., U.S.A., Handke *et al.*, 2015).

To make the enormous corpus of closed access papers retrospectively available to the public might be possible through legal action at an institutional or governmental level. The Dutch Government, for example, has recognized OA as a right, with Dutch citizens capable to make their scientific publications free to access after a ‘reasonable period of time’ ( Open Access NL, 2015). Such steps are further supported by Shavell (2010) and Eger & Scheufen (2012) who ascertained that transition towards an OA model could not be smooth without first undertaking the necessary legislative steps. The position of institutes regarding copyright transfer remains generally unclear. While academics themselves may have little power in broader debates regarding copyright, institutes could claim ownership of the work by invoking their rights under the work made-for-hire doctrine ( Denicola, 2006). However, OA policies at universities generally use a system of non-exclusive rights, presupposing that faculty are the owners of their work and can grant non-exclusive rights to the university for use (for examples of approaches and language used when drafting OA policies, see ( Shieber & Suber, 2016)). Importantly, this means that OA through the ‘Green’ route does not always depend on permission from publishers, and increasingly is becoming dependent on rights retention by authors, through carefully-drafted and widely-supported university policies. While these are positive steps towards making research available for TDM, in light of the potential copyright problems for closed access articles and the fact that not all research is available through institutional Open Access policies, TDM will be easier and legally safer for OA journals. As a consequence, TDM is likely to be more readily applied to OA literature when compared to closed access literature.

## The economic impact of Open Access

### The effect on publishers

Any publisher has to cover operating costs, which are primarily made of (i) article processing charges (APCs), (ii) management and investment costs, and (iii) other costs. Article processing includes editing, proofreading and typesetting, among other things. Management and investment are instead the marginal costs needed to establish and keep the journal running. Other costs include promoting the journal, hosting and infrastructural services, sponsoring conferences, and other services that are extrinsic to research articles themselves. The average production cost for a single research article is estimated to be around $3500–$4000 ( Van Noorden, 2013) but these costs are highly depending on the publisher. For example, Philip Campbell (Editor-in-Chief of *Nature*) stated that his journal’s internal costs were at $20,000–$30,000 per paper ( Van Noorden, 2013), due in part to the high selectivity and rejection rate at *Nature* (i.e., this is an average cost per published paper, and not the production costs associated with publishing a single accepted paper). However, these are at the high end of the cost spectrum, with other journals, such as the *Journal of Machine Learning Research* (JMLR) costing between $6.50–$10 per article ( blogs.harvard.edu/pamphlet/2012/03/06/anefficient-journal/). Other publishers are completely transparent about their direct and indirect production costs, such as Ubiquity Press, which levies an APCs of $500 ( ubiquitypress.com/site/publish/). One possible reason for such variation between journals and publishers is that it is generally unclear whether proposed costs relate to those directly involved in article processing or those required in order for a publisher to ‘break even’ if they receive zero subscription income for an article made OA.

In order to cover those costs and make a profit, closed access publishers charge for access via subscriptions, whereas many OA publishers or journals charge to publish. Due to increased subscription costs, closed access publishing is becoming an increasingly unsustainable business model ( Odlyzko, 2013) with prices estimated to have outpaced inflation at 250% in the past thirty years ( www.eff.org/issues/open-access). This will slowly but surely diminish the scope of access to the scholarly literature as fewer organisations are able to pay such high costs. Only recently has any transparency into the detailed costs of subscriptions been gained by using Freedom of Information Requests to bypass non-disclosure agreements between libraries and publishers ( Bergstrom *et al.*, 2014; Lawson & Meghreblian, 2015). These requests provide the basis for understanding the economics of scholarly communication. For example, Bergstrom *et al.* (2014) found that commercial publishers, including Emerald, Sage, and Taylor and Francis, have prices of ten times the amount of non-profit publishers per citation for PhD-granting institutions. Two potential ways to prevent future retention of an unsustainable model is through decreasing the subscription prices, thereby lowering publishers’ profit margins and the financial burden on subscribers, or through switching to new OA-oriented business models and creating new value. Either way, price transparency will be essential for future bargaining efforts between academic libraries and publishers, and will be of interest to those involved in public policy and scholarly publishing. The concept of transitioning from a subscription-based model to one driven by APCs will be financially appealing to journals that operate with minimal profits or at a loss, and can be a pathway to achieve financial security and long-term journal sustainability. As such, increasing revenues is a strong incentive for OA ( osc.hul.harvard.edu/programs/journal-flipping/public-consultation/4/6/, accessed 26/04/2016).

OA publishing has become associated with a ‘pay-to-publish’ model, whereas around 70% of peer-reviewed OA journals do not charge APCs, according to data from the Directory of Open Access Journals (DOAJ) (see blogs.harvard.edu/pamphlet/2009/05/29/what-percentage-of-open-access-journals-charge-publication-fees/ and citesandinsights.info/civ16i4.pdf). However, approximately 50% of all articles published in peer-reviewed OA journals are published in APC-based venues ( Crawford, 2015; Laakso & Björk, 2012; Walters & Linvill, 2011). Authors paying to publish can be viewed as a fundamental conflict of interest for researchers. Nonetheless, this payment model has proven itself to function properly when editorial decisions are separated from the business-side of the publisher (i.e., editorial independence), removing the problem of ‘publication-bribery’. Additionally, many journals have always levied charges for to cover the costs of publishing regardless of OA; for example, *PNAS* charges $1225 per regular research article (with an additional $1350 for OA; pnas.org/site/authors/fees.xhtml), and *Cell* charges $1000 for the first colour figure and $275 for each subsequent one ( cell.com/cell/authors; as of April 2016). Therefore, equating OA with ‘pay-to-publish’ is actually a bit of a misnomer, as several closed journals charge to publish and many open journals do not. Furthermore, many publishers (e.g., *PLOS*, *PeerJ*), as well as many learned societies, operate fee waiver schemes for researchers unable to obtain funds to cover publication fees.

For those OA publishers implementing a pay-to-publish model, around 68.8% of publishers offer fee waivers to low- and middle-income countries ( Lawson, 2015), while other journals offer fee discounts often given in lieu of total fee waivers. Solomon & Björk (2012)  investigated the sources of funding used by authors for APCs, indicating that these are highly variable across academic disciplines. For example, while 45.5% of authors in Health Sciences, Biology and Life Sciences use grant or contract funding as source for APCs, only 10.4% use this in Business and Economics, with 45.8% coming from personal funds. Other sources include national funding bodies, and discretionary funds administered by institutions, as well as institutional funds specifically in place to support OA policies (see also Dallmeier-Tiessen *et al.*, 2011). Sources for APCs are also highly variable depending on the per capital GNP of the authors’ country, as well as the size of the APC ( Solomon & Björk, 2012). According to MacKie-Mason (2016), one potential outcome of authors seeing the price of APCs and securing funding for them is that authors may begin to take the price of APCs into account (in addition to other factors such as prestige and topic) when selecting a journal for their research output, which may drive market competition and could as a consequence lower the price of APCs. However, a potential negative consequence of an increasingly APC-driven model of OA is that some researchers may struggle to procure funds in order to publish and conform to mandates at different levels. This might impact early-career researchers and those working in fields were research grants and publishing fees are more difficult to obtain.

Subscription-based publishers still frequently produce print versions of their journals, which increases production costs, potentially to justify charging for readership or to satisfy a small demographic who prefers this mode of reading. After all, subscriptions to print journals make sense and, if large-scale printing is still in place, simply transferring this idea to the digital versions creates continuity. Print versions are accompanied by logistical costs to print and ship each issue, but these are partially offset with reprint orders, additional charges for colour figures, and print-based advertising. For some of the largest subscription-oriented publishers the annual net profit on investment reaches up to 40 percent, which makes academic journal publishing highly lucrative for investors ( Satyanarayana, 2013), further increases investment to sustain this type of publishing model, and allows maintenance of an oligopoly ( Larivière *et al.*, 2015).

OA publishers only publish digitally and have opened up avenues for innovation. For example, *PeerJ* has introduced a wholly different OA business model, where readers pay nothing to access articles, but authors pay a membership fee once to publish for a lifetime. The Open Library of Humanities (OLH) is another innovative business model in which libraries pay a small fee to support OLH and scholars are able to publish for free (subscription for publishing rather than subscription for access); this support also enables the OLH to help journals transition from a subscription model to OA (for example, the recent case of *Lingua*; timeshighereducation.com/research-intelligence/open-library-humanities-aims-flip-journals-open-access). Library publishing has also developed in response to the OA movement; in this model, academic libraries begin publishing operations in the interest of providing added value to their patrons and contributing to the growth of knowledge ( librarypublishing.org). In terms of innovating in the publishing platform itself, *eLife* have introduced the Lens as a novel way of viewing research articles online ( lens.elifesciences.org/about/), and *F1000Research* has introduced so-called ‘living figures’ to enable researchers to interact with data underlying research findings (e.g., Colomb & Brembs, 2015).

With this innovation comes massive scope for reducing the costs associated with publishing through implementing more efficient procedures. In this case, costs are reduced by eliminating the need for type-setting and copy-editing, with web-hosting costing only $15/year, and a total operating cost of between $6.50–$10.50 per article. Other platforms such as ARPHA offer an end-to-end XML-based publishing service, utilised by publishers including Pensoft, with a more efficient and integrated publishing workflow, which should highlight and reduce the real costs of publishing. In addition, OA has the potential to increase the speed of publication, as seen in journals like *eLife* and *PeerJ* ( blog.dhimmel.com/plos-and-publishing-delays/), which combined with ‘pre-print’ servers like *biorXiv* and platforms that offer post-publication peer review like *Research Ideas and Outcomes* ( riojournal.com/), *F1000 Research*, and *ScienceOpen* ( www.scienceopen.com/), can exceptionally accelerate the speed of research communication. Such innovations add value to the research communication process (contrary to services such as paying to print colour figures) and represent just several cases of innovation across the publishing ecosystem. One can imagine that publishing costs in OA journals become dependent on the value added on a per-article basis, which can help reshape and improve scholarly communication. As such, making publication costs dependent on the value added aligns the interests of publishers with those of scholars, where improving the quality of the process of scholarly communication is the end goal. The motivation behind this could come from the currently available data, which suggest that hybrid publishing options offered by traditional publishers, while being of higher cost due to supposed prestige, provide a much lower overall quality publishing process ( blog.wellcome.ac.uk/2016/03/23/wellcome-trust-and-coaf-open-access-spend-2014-15/). It is noteworthy that in spite of the higher costs of hybrid publishing compared to ‘pure’ or ‘born’ OA publishing, some reports, such as the highly influential and somewhat controversial Finch Report in the UK ( www.researchinfonet.org/publish/finch/), favoured the former model and high-priced Gold OA over a Green model.

### The effect on non-publishers

**Figure 3.**
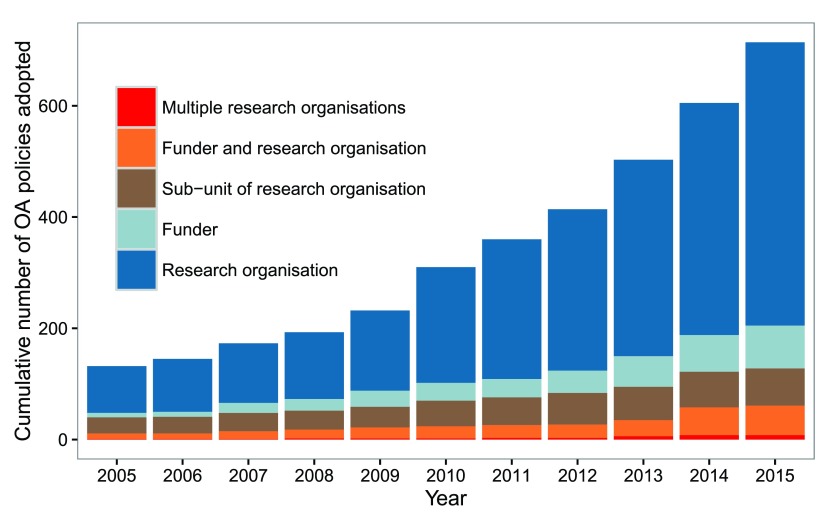
Cumulative number of Open Access policies adopted by multiple research organisations, funder and research organisation, sub-unit of research organisation, funder, and research organisation. Figures are given at the beginning of each year. Source: ROARMAP, accessed March 2016.

The implementation of OA models has implications beyond the publishing industry in terms of economics. Research funding comes from multiple sources, including national funding agencies and industries, as well as private funders. Much primary research actually takes place outside of academia, inside R&D departments; if R&D in the private sector can access more research findings, this will ultimately benefit the public interest as well. A report from 2004 by Arzberger and colleagues into the scientific, social and economic development of access to research results concluded that access should be promoted to the largest extent possible. According to this report, access to research results can only be responsibly restricted in the case of national security, privacy, or those involving IP rights of the authors ( Arzberger *et al.*, 2004). A major principle underlying this is the ownership of research results: publicly funded research and data are public goods and because they have been produced in the public interest they should be considered and maintained as such. Indeed, such a principle has become one of the focal rallying points of the global OA movement. Appropriate licensing and accessibility can influence re-use through commercialization, and can empower citizens and industry to recognize great economic benefits. This apparently resonates with many organisations, as indicated by the increased numbers of OA policies on a global basis (see Figure 3).

With access to scholarly articles, entrepreneurs and small businesses can accelerate innovation and discovery, which is advantageous for advancing the ‘entrepreneurial state’ ( Mazzucato, 2011). Access to research results has clear advantages for a range of industries and can help stimulate regional and global economies. Increased access to research results has been associated with considerable increases of return on financial investment ( Beagrie & Houghton, 2014). Furthermore, OA facilitates collaborations between publishers and industrial partners to leverage the potential of structured information networks for advanced data mining projects, such as that recently announced between IBM Watson and PLOS ( Denker, 2016). One of the major driving forces behind the development of OA in the UK on a national level, the ‘Finch Report’, also concluded that OA was an essential source for information and innovation to the civil service, commercial sectors, small- and medium-sized enterprises (SMEs), and the general public ( www.researchinfonet.org/publish/finch/).

Taking UK cancer research as one high impact case study, there is substantial evidence for the economic benefit of OA. In 2011–12 prices, the total expenditure on research relating to cancer in the period of 1970–2009 was £15 billion ( Glover *et al.*, 2014). 5.9 million quality adjusted life years were gained from the prioritized interventions in 1991–2010, of which the net-monetary benefit was an estimated £124 billion (i.e., eight-fold return on investment). However, only 17% of the annual net-monetary benefit was estimated to be attributable to research performed in the UK ( Glover *et al.*, 2014), suggesting that 83% of the economic return on cancer research is drawn from research from non-UK sources. Another example is from the area of environmental impact assessments, where Vickery (2011) has shown that OA to R&D results could result in recurring gains of around €6 billion per year. As such, opening up research for global access rather than localized and restricted use has the potential to increase the economic return, as demonstrated with the cases on cancer research and environmental impact assessments.

### The price of Open Access

The question of the current publication cost is difficult and confounded by estimates of the total global publishing costs and revenue. Data provided by Outsell, a consultant in Burlingame, California, suggest that the science publishing industry generated $9.4 billion in revenue in 2011 and published around 1.8 million English-language articles. This equates to an approximate average revenue per article of $5,000. A white paper produced by the Max Planck Society estimated costs at €3,800–€5,000 per paper through subscription spending, based on a total global spending of €7.6 billion across 1.5-2 million articles per year in total ( Schimmer *et al.*, 2015). Other estimates suggest that the total spending on publishing, distribution and access to research is around £25 billion per year, with an additional £34 billion spent on reading those outputs, a sum which equates to around one third of the total annual global spending on research (£175 billion; Research Information Network, 2008).

Such high costs are at odds with alternative estimates of the cost of OA publishing. For example, the Scientific Electronic Library Online ( *SciELO*) is a pan-Latin American bibliographic database, digital library, and co-operative electronic publishing model of OA journals. It is estimated that their costs are between $70 and $600 per OA article depending on the services provided ( Brembs, 2015). OA now dominates the Latin American publishing landscape, with an estimated 72–85% of articles now with full text OA articles publicly available ( www.sparc.arl.org/news/open-access-latin-america-embraced-key-visibility-research-outputs). Furthermore, in countries such as Brazil, higher quality journals are more likely to be published OA ( Neto *et al.*, 2016), implying that low-cost, high quality, and OA can all co-exist. Even more extreme estimates of the cost of OA come from Standard Analytics, who suggested the absolute minimum per-article costs of publishing could fall to between $1.36 and $1.61 with sufficient cloud-based infrastructure ( Bogich *et al.*, 2016). However, it is likely that this estimate under-emphasizes marginal costs that are beyond a per-article cost basis. However, what is clear from these analyses is that OA has the opportunity to become a cost-reducing mechanism for scholarly publishing. Open Journals System (OJS), an open source software available for anyone to use and download without charge, is another example of this. Additionally, researcher-led initiatives such as the recently launched *Discrete Analysis* have costs that average around $30 per article, with no cost to authors or readers, and utilise the infrastructure offered by the arXiv to keep costs low ( discreteanalysisjournal.com).

In her article, Sutton (2011) argued that current scholarly journals are digital products and that as such they are driven by very different economic principles and social forces than their print ancestors. Based on Anderson (2013b), the author made the case that changes in both the delivery of scientific content and in publishers’ business models was inevitable when journals moved online. Sutton (2011) considered that scientific literature is no different from other digital products with respect to distribution costs and as such it is no exception to the ‘zero is inevitable’ rule of pricing.

## The societal impact of Open Access

OA to the scholarly literature does not just benefit academics, but also has wider impacts on other domains in society. It makes research available to anyone with an Internet connection who has the ability to search and read the material. Therefore, it transcends academic affiliation and supports sustainable lifelong learning. Examples of groups who might benefit most from OA include citizen scientists, medical patients and their supporting networks, health advocates, NGOs, and those who benefit from translation and transformation (e.g., sight-impaired people). In theory, OA affects anyone who uses information, and opens up possibilities for knowledge to be used in unexpected, creative and innovative ways, far beyond the mainstream professional research.

Access to knowledge has been called a human rights issue, considering it is included in Article 27 of the United Nations Declaration of Human Rights. Willinsky (2006) has argued that " *Access to knowledge is a human right that is closely associated with the ability to defend, as well as to advocate for, other rights.*". This is not only true for access to knowledge from research that could save human lives, but also, as argued by Jacques Derrida, to the right of access to philosophy and the humanities disciplines that stem from it. Derrida writes about the field of Philosophy, " *No one can forbid access to it. The moment one has the desire or will for it, one has the right to it. The right is inscribed in philosophy itself* " ( Derrida, 2002).

Society’s ability to make research publicly accessible supports the long-term interest and investment in research. Citizens support research through taxes and therefore one could argue that efforts to support public access should be a fundamental part of the research process. While OA is not a solution to all aspects of research accessibility (e.g., filtering and language barriers, connectivity barriers and disability access remain continuing issues to be addressed; cyber.law.harvard.edu/hoap/Open_Access_(the_book)), it most certainly increases accessibility greatly and at the same time allows innovations to remove other barriers (e.g., OA articles can be freely translated to address language barriers and can be changed to different formats to accommodate screen readers). Anecdotal evidence suggests that public access to research is required from a range of public spheres ( whoneedsaccess.org/). Nonetheless, the fact that access to knowledge continues to be prohibited in fields like public health should be of major concern to all stakeholders engaged in academic publishing.

In addition to professional research by, for example, academics, there is the dimension of citizen science. In citizen science, the broader public participates in the research process itself and will have an increased interest in accessing previous research. Numerous projects such as Galaxy Zoo, Zooniverse, Old Weather, Fold It, Whale FM, Bat Detective, and Project Discovery, are all different initiatives in which citizens publicly and openly engage with research. These initiatives introduce new ways of knowledge creation and these groups also require thorough access to actually be able to do non-redundant research. Citizen science forms part of the societal case for OA, because it clearly indicates that anyone can be actively engaged with research, and not only professional scientists.

Some traditional publishers and some academics have argued that public access to research is not required because research papers cannot be understood by non-specialists ( cyber.law.harvard.edu/hoap/Open_Access_(the_book) - see Section 5.5.1). However, citizen science initiatives already indicate the general public *is* interested in and understands the research. Whereas this understanding and engagement is highly variable, and strongly dependent on a range of extrinsic and intrinsic factors, the fact that a high level of public interest in science already exists is of relevance. These publishers and academics argue that specialization is a sufficient reason for confining access to professional research bodies through subscriptions. Such statements conflate a lack of desire or need for access with the denial of opportunity to access research, and makes false presumptions about the demand in access to the literature (i.e., unmet and unknown demand). Importantly, OA provides access to everyone who potentially needs or wants it, without making explicit and patronising statements or guesswork about who needs or deserves it. As Peter Suber says in his 2012 book: "The idea [of OA] is to stop thinking of knowledge as a commodity to meter out to deserving customers, and to start thinking of it as a public good, especially when it is given away by its authors, funded with public money, or both" (page 116). Isolated incidents such as the crashing of servers of Physical Reviews Letters upon the ‘Gravitational Waves’ announcement and OA publication (Feb, 2016; Abbott *et al.*, 2016) indicate that there are cases of extreme public interest in science that closed access would only impede. Moreover, one out of four people seeking medical information have hit a paywall at least once ( pewinternet.org/2013/01/15/information-triage/). Claims that only experts can and should read research articles does little to break down the ‘ivory tower’ perception that still pervades academia, and undermines the enormous amounts of resources invested in science communication and public engagement activities. Such perceptions run counter to the idea of access to knowledge as a right, retaining it as a privilege based on financial or academic status.

### Open Access in developing countries

The arguments outlined above form the basis for democratic and equal access to research, which come to light even stronger in the developing world. For low- and middle-income countries (LMIC), OA publishing breaks traditional financial barriers and allows unrestricted, equal access to scholarly information to people all over the globe. Due to the high prices of journal subscriptions, developing countries struggle with access just as in developed countries, but to a greater extent and consequently with greater negative repercussions. For example, a research paper from 1982 that indicated why Liberia should be included in the Ebola endemic zone was unknown to Liberian officials in the 2014 Ebola outbreak ( Knobloch *et al.*, 1982); the paper was published behind a paywall, drastically reducing its discoverability. Even though the result is available in the abstract of the paywalled article, assessing the truth of the result certainly requires access to the full research article. In general, lack of access can have major deleterious consequences for students and researchers, in that they do not have sufficient material to conduct their own primary research or education.

OA provides a mechanism to level the playing field between developed and developing countries. This increases fair competition and the scientific potential of the developing world ( Chan *et al.*, 2005). This aspect is linked to the wider issue of open licensing, which is essential for effective marketing of medicines and medical research in developing countries ( Flynn *et al.*, 2009), and justifies the necessity of OA in the wider context of social welfare. Developing countries clearly acknowledge the need for access and as such have launched many repositories to increase access with self-archiving of research articles. In 2014, over 100 institutions in Africa launched a network of over 25 fully-operational OA repositories in Kenya, Tanzania and Uganda ( www.ubuntunet.net/april2014#researchrelevant). Such developments suggest that African nations are leaning more towards a Green model of OA adoption.

The shift from a ‘reader pays’ to a pre-publication fee model (often conflated with ‘author pays’; see subsection ‘The effect on publishers’) with OA potentially limits its adoption in developing countries. The pay-to-publish system is a potentially greater burden for authors in developing countries, considering that they are not used to paying publication costs, and funding systems for OA are not as well-established as those in the Western world. Publication fees present an even greater relative burden ( Matheka *et al.*, 2014) given that they can often exceed a monthly salary for researchers. This has been at least partially mitigated with waiver fees for authors from developing countries and additional provisions in research grants, and around 70% of peer reviewed OA journals are fee-free. In November 2015, Research4Life ( research4life.org) and DOAJ announced a working partnership that will help to ensure that the Research4Life users will have access to the largest possible array of OA journals from publishers with a certain quality standard. While Research4Life does not directly cover OA publication costs, a lot of publishers propose full or partial waivers if they are based in countries eligible by Research4Life. However, determining which countries qualify for access to scientific journals through these programs, and which journals they are provided access to, is a fairly closed process. They are also not entirely stable, as publishers can opt out of the initiative, or be selective about which countries they choose to serve. In 2011, publishers withdrew free access to 2500 health and biomedical journals for Bangladesh ( Kmietowicz, 2011) through the HINARI programme. While access was subsequently reinstated, this demonstrates that such initiatives are not an adequate replacement for full OA ( Chatterjee *et al.*, 2013). Despite these programs purporting to provide essential articles to researchers in poor nations, they exclude some developing countries (e.g., India) and limit access to researchers who work in registered institutions.

Initiatives such as the Journals Online Project developed by INASP (International Network for the Availability of Scientific Publications; inasp.info/en/) has helped to develop a number of online OA platforms in the Global South. These were launched in 1998 with the African Journals Online (AJOL) platform, a project currently managed in South Africa. More recently, INASP have set up Latin American Journals Online (LAMJOL) which hosts journals in El Salvador, Honduras, and Nicaragua. In Asia, Bangladesh Journals Online (BanglaJOL), Nepal Journals Online (NepJOL), and Sri Lankan Journals Online (SLJOL), all facilitated through INASP, continue to develop and now around 95% of their articles are full-text Open Access. As mentioned previously, improved access should not be limited to professional researchers only, considering that there is also global interest from the broader public, including health professionals.

### Deceptive publishing practices

One negative effect of OA comes from entities that attempt to profit by exploiting the pay-to-publish system used by many OA publishers. These publishers operate a sub-category of OA journals known as vanity presses, predatory publishers ( Beall, 2012) or pseudo-journals ( McGlynn, 2013). These journals, referred to in this work as ‘deceptive publishers’, seem to be in the scholarly publishing business primarily to collect publication fees (i.e., APCs) in exchange for rapid publication without formal peer-review. Beall (2015) has defined a list of criteria for identifying deceptive publishers and an index of publishers and individual journals that meet these criteria is continuously updated ( scholarlyoa.com).

While not all scholars and advocates agree with the criteria proposed by Jeffrey Beall (who controversially describes the OA movement as "an anti-corporatist movement that wants to deny the freedom of the press to companies it disagrees with" ( Beall, 2013)), there are several factors that many agree on to identify a deceptive publisher, but these factors are not clear-cut indicators of deceptive publishing. One such indicator is that deceptive publishers tend to charge low publication fees ( Xia, 2015), most below $100 and few charge more than $200. However, while this is a trait of almost all deceptive publishers, the reverse is not necessarily the case. For example, a single-authored paper with *PeerJ* would cost $99, but this is not a deceptive publisher. On the contrary, the average publication fee of journals indexed in the Directory of Open Access Journals (DOAJ) is around $900–$1,000 ( Solomon & Björk, 2012) and leading universities in the UK and Germany pay on average $1,200–$1,300 per article ( Schimmer *et al.*, 2015). The editorial and peer-review aspects of deceptive publishers are either non-existent or suspect; they also falsely claim to have ratings such as a Journal Impact Factor and to be indexed in major databases such as Scopus ( Djuric, 2015). Editors from these journals solicit articles that have no relation to the topic of their journal and do not send the manuscripts out to be properly peer-reviewed ( Bowman, 2014).

The problem of deceptive publishers in OA seems to highly affect countries where the academic evaluation strongly favors international publication without further quality checks ( Shen & Björk, 2015). Xia *et al.* (2015) collected and analyzed the publication record, citation count, and geographic location of authors from the various groups of journals. Statistical analyses verified that deceptive and non-deceptive journals have distinct author populations: authors who publish in deceptive journals tend to be early-career researchers from developing countries with still little publishing experience. The spatial distribution of both the deceptive publishers and those authors who submit in pseudo-journals is highly skewed: Asia and Africa contributes three quarters of authors ( Xia *et al.*, 2015) and Indian journals form the overwhelming proportion of deceptive publishers ( Xia, 2015). An interesting finding is the very low involvement of South America, both among deceptive publishers (0.5%) and corresponding authors in deceptive journals (2.2%). The OA infrastructure in Latin America is different compared to other developing countries, which reveals a possible reason for this asymmetric situation. Latin American journals and universities are engaged in OA publication models at a higher degree than other regions ( Alperin *et al.*, 2011). As a result, scholars from this region are not only more aware of OA issues, but they have more options for publishing OA than those from other regions ( Alperin *et al.*, 2011). Moreover, SciELO ( Packer, 2009) and the creation of Latin American databases ( Alonso-Gamboa & Russell, 2012) have played a tremendous part in this process by bringing recognition and a good reputation to publishing outlets in Latin America.

Considerable attention is given to the subject of deceptive publishers, who have become conflated with the OA movement in general to the detriment of genuine OA publishers. For example, a ‘sting’ operation that outed bad peer-review instead got misinterpreted as bad peer-review in OA journals ( Bohannon, 2013), but was probably more indicative of issues to do with the traditional closed and over-burdened system of peer review ( scilogs.com/communication_breakdown/jon-tennant-oa/). Overall, the deceptive publisher phenomenon is one major negative aspect that spawns many misconceptions and misgivings about publishing OA. Recently launched industry-led initiatives such as "Think, Check, Submit" ( thinkchecksubmit.org) provide a checklist to help researchers identify trustworthy journals, and will likely be a pivotal tool in combating deceptive publishers.

## Open Access and Open Science

OA exists in a constantly evolving scholarly research ecosystem and the proliferation of "open" as a description of scientific activities has caused some confusion about what the term "open" means (for a more comprehensive discussion, see Pomerantz & Peek (2016)). As such, it is important to note how it is interconnected to other facets of the scholarly communication system. Here, we discuss the implications that the transition to OA has on developments in the broader context of Open Science (or Open Research).

### Open Access and Open Data

The overall movement of OA has become conjoined with the drive for Open Data. Data sharing is fundamental to scientific progress, because data lead to the knowledge generated in research articles. Furthermore, data sharing has recently become a common requirement, together with OA, for both research funding and publication. The data sharing policy from PLOS illustrates the high degree of overlap between OA and Open Data; authors of articles published in PLOS are required to share the data except if they have valid reasons not to (i.e., an opt-out system; journals.plos.org/plosone/s/data-availability). Many publishers, NGOs, and research funders have recently come together to commit to free research sharing in times of public health emergency, catalysed by the current Zika health threat ( http://www.wellcome.ac.uk/About-us/Policy/Spotlight-issues/Data-sharing/Public-health-emergencies/index.htm). It is noteworthy that some of the largest publishers, including Wiley, Taylor and Francis, and Elsevier (with the exception of the journal *The Lancet*) did not commit to research sharing during ongoing or future public health crises (as of May, 2016).

The benefits of Open Data are diverse, including a citation advantage. Combined with the citation advantage for OA articles, providing data alongside publications can increase citations on average by 30% ( Piwowar & Vision, 2013) and up to 69% ( Piwowar *et al.*, 2007), but this evidence is entirely field-dependent (e.g., Dorch *et al.*, 2015)). Below we cover seven additional benefits of Open Data.

First, data sharing enhances reproducibility, a crucial aspect in a time where some scientific domains appear to have problems with reproducibility (e.g., Open Science Collaboration, 2015). Several factors could form the basis for this ‘crisis’, such as an overemphasis on novelty instead of rigour, selective reporting of results, an overemphasis on statistical significance, and insufficient documentation of the research methods. Publicly sharing data, code, and materials can certainly alleviate issues with reproducibility. This is especially pertinent in the modern sciences, where a substantial proportion of published results draw on quantitative experiments and computer simulations. As such, it is largely impossible to reproduce these experiments as they become more complex and the associated datasets increase in complexity. When full access to the data, metadata, and the code used to produce the ultimate results are provided alongside publication, this greatly improves reproducibility.

Second, publicly available data can be used to stimulate innovations, such as new analytical methods. An excellent example of this is provided by the neuroimaging OpenfMRI project, where shared data have been used to examine the effects of different processing pipelines on analysis outcomes ( Carp, 2012) and test new methods to characterize different cognitive tasks ( Turner & Laird, 2012). Another good example is the Protein Data Bank (PDB) ( Berman *et al.*, 2000), a project which has enabled the re-use of the primary structural data and opened up new avenues of research, despite the latter not being expected.

Third, data sharing enables new research questions that can only be answered by combining datasets which now remain separated. Analyzing vast volumes of data can yield novel and perhaps surprising findings. This allows for integrated research hypotheses on the underlying processes behind the original data and observations. Exploratory approaches to large datasets can be seen as hypothesis generating tools, which later drives experimental testing to confirm or disprove these hypotheses ( Wagenmakers *et al.*, 2012).

Fourth, the realization that data will ultimately be shared and visible to the community provides a strong incentive for researchers to ensure they engage in better data documentation and, therefore, research methods. For example, the willingness to publicly share data has been associated with fewer statistical errors in the final research article ( Wicherts *et al.*, 2011).

Fifth, public data sharing provides a digital backup for datasets, protecting valuable scientific resources. Moreover, a considerable amount of data produced every day does not ultimately lead to publication and often remain hidden. Such data might remain in a hidden file-drawer despite being valid, creating a systematic bias in the information available. Public data sharing opens this file-drawer and, consequently, allows independent assessments of whether the data are valid or not.

Sixth, sharing data can certainly reduce the cost of performing research. A file-drawer has been indicated to greatly reduce the efficiency of research in detecting effects ( van Assen *et al.*, 2014). Open Data, as such, discourages redundant data collection (i.e., data that have been already collected but never made publicly accessible) and simultaneously allows researchers to better approximate what is happening in their fields. This will have a large effect on research costs, resulting in savings that can be then be used for more productive research goals.

Finally, and tightly connected with the sixth point, Open Data potentially has a great economic value. For example, Open Data creates jobs for analysis and re-use of these data Capgemini (2015), and contributes to additional value of products and services in major sectors ( Manyika *et al.*, 2013), ad well as benefits users of these data rich services ( Stott, 2014).

### Open Access and Open Science

Beyond OA and Open Data lies a more integrated approach to research, referred to more broadly as Open Science (i.e., Science 2.0, Open Scholarship). According to the European Commission’s Horizon 2020 programme, Open Science is defined as " *The transformation, opening up and democratisation of science and research through ICT, with the objectives of making science more efficient, transparent and interdisciplinary, of changing the interaction between science and society, and of enabling broader societal impact and innovation*". Consequently, we see OA as only one of the multiple challenges currently facing the ‘open transformation’ of the scholarly publishing system ( Watson, 2015), and OA should therefore be considered in the wider contexts and complimentary domains of research transparency and open source.

As Kriegeskorte *et al.* (2012) pointed out, OA is now widely accepted as desirable and becoming a reality in many academic spheres. However, the second essential complementary element to research, evaluation, has received less attention despite the large amount of research that has been done to document its current limitations ( Benos *et al.*, 2007; Birukou *et al.*, 2011; Ioannidis, 2005; Ioannidis, 2012a; Ioannidis, 2012b; John *et al.*, 2012; Nosek & Bar-Anan, 2012; Simmons *et al.*, 2011).

Open evaluation, an ongoing post-publication process of transparent peer review and rating of papers, promises to address the problems of the current assessment systems Kriegeskorte *et al.* (2012), as well as increasing the overall quality of the peer review process. As such, ongoing assessments of the development of OA must also consider the broader impact and concurrent changes to the peer review system ( van Rooyen *et al.*, 1999; Wicherts, 2016; Leek *et al.*, 2011). Some assessment methods, such as the Research Excellence Framework (REF) in England and administered by HEFCE, have already made OA a core feature of evaluation in that all research papers submitted to the REF must be archived in an institutional or subject repository ( www.hefce.ac.uk/pubs/year/2014/201407/). While it is too early to evaluate the impact of this policy, by tying OA compliance with research evaluation we might expect to see a national shift towards large-scale OA adoption. At the very least, such a combination is generating increasing interest and awareness about OA among researchers, increasing usage of institutional repositories, and increasing demand for funding for APCs ( Tate, 2015).

Future research regarding better ways to improve scholarly communication will be instrumental in providing evidence to support the transformation of the publishing system and design new alternatives ( Buttliere, 2014; Ghosh *et al.*, 2012; Kriegeskorte *et al.*, 2012; Pöschl, 2012), which will draw heavily upon on open publishing framework driven by developments and newly emerging models in OA. Finally, consideration of Open Science and OA will be important inclusions in evolving research standards such as the Transparency and Openness Promotion (TOP) guidelines ( https://cos.io/top/) .

## Conclusions

This article provides an evidence-based review of the impact of OA on academy, economy and society. Overall, the evidence points to a favorable impact of OA on the scholarly literature through increased dissemination and re-use. OA has the potential to be a sustainable business venture for new and established publishers, and can provide substantial benefits to research- and development-intensive businesses, including health organisations, volunteer sectors, and technology. OA is a global issue, highlighted by inequalities between developing and developed nations, and largely fueled by financial disparity. Current levels of access in the developing world are insufficient and unstable, and OA has the potential to foster the development of stable research ecosystems. While deceptive publishing remains an ongoing issue, particularly in the developing world, increasing public engagement, development of OA policies, and discussion of sustainable and ethical publishing practices can remove this potential threat.

For libraries, universities, governments, and research institutions, one major benefit of lowering the cost of knowledge is the availability of extra budget that can be reallocated for other purposes. For researchers themselves, OA can increase their audience and impact by delivering wider and easier access for readers. For publishers, promoting OA is an answer to the desires and the needs of their research communities. Furthermore, subscription-based publishers have (partly) answered the call of the increasing global demand for OA, by giving their green light to author self-archiving ( Harnad *et al.*, 2008), as well as by establishing numerous ‘hybrid’ OA options. In an author survey, Swan & Brown (2004) reported that the vast majority of their sample indicated that they would self-archive willingly if their employer (or funding body) required them to do so. Similarly, in a study by Swan & Brown (2005) the vast majority of researchers (81%) indicated that they would comply with mandates that made OA a condition of funding or employment. There is evidence that many funders and research organisations are moving in this direction: since 2005, the number of policies supporting OA publishing increased steadily, and there is similar growth in the number of institutional rights-retention policies. Consequently, it is now the responsibility of researchers to ensure OA to their publications either by choosing the Green or the Gold road, and for public research funders to employ policies that are in the best interests of the wider public while considering the financial sustainability of the scholarly publishing ecosystem.

The fact that OA impacts upon such a diverse range of stakeholders, often with highly polarised and emotional viewpoints, highlights the ongoing need for evidence-informed discussion and engagement at all levels. This is especially the case for research communities, who have exceptionally diverse perspectives about OA and in particular how it interacts with ‘quality’ and ‘prestige’ in publishing ( Schroter & Tite, 2006; Schroter *et al.*, 2005). As Peter Suber, a leading voice in the OA movement, stated ( dash.harvard.edu/handle/1/4391169).

" *TA [toll-access] publishers are not the enemy. They are only unpersuaded. Even when they are opposed, and not merely unpersuaded, they are only enemies if they have the power to stop OA. No publisher has this power, or at least not by virtue of publishing under a TA business model. If we have enemies, they are those who can obstruct progress to OA. The only people who fit this description are friends of OA who are distracted from providing OA by other work or other priorities.*"

Therefore, OA supporters should focus their efforts on working for new models and systems rather than trying to undermine or punish the existing ones. OA remains only one of the multiple challenges that the scholarly publishing system is currently facing. As highlighted in this review, the empirical evidence for OA is overwhelmingly positive, but further research is certainly required to move from investigating the effects of OA to researching the broader effects of Open Science. In particular, OA must be considered in the future to more broadly regarding the adverse effects of a system of journal-based research assessment ( Brembs *et al.*, 2013), and the development of scholarly communication systems that are sustainable for, and in the best interests of, the commons.
