# Supplementary material for: The academic, economic and societal impacts of Open Access: an evidence-based review
Source: F1000Res. 2016 Sep 21;5:632. Originally published 2016 Apr 11. [Version 3] doi: 10.12688/f1000research.8460.3 (PMC4837983; doi:10.12688/f1000research.8460.3)
Supplement: Peer review history

# Peer review: Referee response for version 3

**DOI:** 10.5256/f1000research.10376.r16583

**Published:** 2016-09-26

**Authors:** Peter Suber

**Affiliations:** 1 Berkman Center for Internet & Society, Harvard University, Cambridge, MA, USA

I'm commenting on this sentence from the first paragraph of Version 3 of the article:

 The Green route is also enabled through author rights retention, in which authors pre-emptively grant non-exclusive rights to their institutions before publishing any works. The institution then has the ability to make articles by these authors OA without seeking permission from the publishers (e.g., this is the case of the Dutch Taverne amendment that has declared self-archival of research after ‘a reasonable period of time’ a legal right ( Open Access NL, 2015)).

 ​The authors added the bulk of this at my request in an earlier comment. I'm glad they did, but it's still only partially true. It's true that universities can adopt rights-retention OA policies that make it unnecessary to seek permission from publishers. But it's not true that Dutch Taverne amendment is an example. It's not a university policy, but legislation. It's legislation that gives authors permission for green OA regardless of the contracts they signed, and regardless of the rights they might have retained through a university policy. It's a very good idea and I recommend it everywhere. (There is already a similar law in Germany, and one is now emerging in France.) But what the authors need here is an example of a university rights-retention OA policy, or a thorough explanation of this kind of OA policy.

 I'm not seeking a citation to my own university's activity, though it adopted the first OA policies of this kind. Nor am I seeking a citation to the good-practices guide that I maintain with Stuart Shieber, though it's the standard reference on this kind of policy. In fact, I was reluctant to follow up the authors' response to my prior suggestion because I didn't want to appear to seek additional citations. 

 But since I've gone this far, I'll mention these two sources anyway:

 The Harvard open-access policies

https://osc.hul.harvard.edu/policies/

 ​Good practices for university open-access policies

http://bit.ly/goodoa 

 Of the two, I'd recommend the second in this situation. But even if the authors include no citation on this point, at least they should stop citing the Dutch law as an example, and treat it separately as another path to the same goal.

I have read this submission. I believe that I have an appropriate level of expertise to confirm that it is of an acceptable scientific standard, however I have significant reservations, as outlined above.

# Peer review: Referee response for version 2

**DOI:** 10.5256/f1000research.9609.r14274

**Published:** 2016-08-25

**Authors:** Gwilym Lockwood

**Affiliations:** 1 Neurobiology of Language Department, Max Planck Institute for Psycholinguistics, Nijmegen, Netherlands

I have no further comments; my points or concerns have been addressed, and other issues are highlighted in further depth by other reviewers.

I have read this submission. I believe that I have an appropriate level of expertise to confirm that it is of an acceptable scientific standard.

# Peer review: Referee response for version 2

**DOI:** 10.5256/f1000research.9609.r14275

**Published:** 2016-06-27

**Authors:** Peter Suber

**Affiliations:** 1 Berkman Center for Internet & Society, Harvard University, Cambridge, MA, USA

In the first version I reviewed, you said, "This [green] route is dependent on journal or publisher policies on self-archiving (sherpa.ac.uk/romeo)."

 That was untrue or incomplete and I offered this comment: It overlooks rights retention. Some individual authors retain enough rights to authorize green OA on their own. While this may be fairly rare, rights-retention OA policies at universities are increasingly common. More than 80 institutions in North America, Europe, Africa, and Asia have now adopted rights-retention OA policies. Under these policies, the faculty grant non-exclusive rights to their institution before they sign future publishing contracts. The institution then has permission to make those future articles OA without having to seek permission from publishers. (The institutions also grant the same non-exclusive rights back to authors.) For more detail on rights-retention OA policies, see Stuart Shieber and Peter Suber, "Good Practices for University OA Policies."

 http://bit.ly/goodoa

 You revised the text in a way that missed my point and misstated my position: "While academics themselves may have little power in debates regarding copyright, institutes could claim ownership of the work they likely already own by invoking their rights under the work made-for-hire doctrine (Denicola, 2006). However, it is difficult to imagine researchers favoring university-held rather than journal-held copyright, and a system of non-exclusive rights is preferred, as is reflected in OA policies and OA journals (Suber, 2012)."

 Here's the main point: More than 80 universities around the world have adopted rights-retention OA policies. These policies are adopted by faculty votes, not administrative edicts. At these institutions the rights needed to authorize OA are not seized from faculty by the institution, or claimed by the institution through work-for-hire. These policies presuppose that the rights initially belong to faculty, not the institution. If the institution is to exercise them, faculty must voluntarily grant them. (There are details we needn't go into here, for example, that we're only talking about non-exclusive rights, and that these policies generally include waiver options when faculty don't want the institution to have rights to a given work.) At Harvard, which pioneered this type of policy, four of the school-level votes were unanimous. In short, it's not at all "difficult to imagine researchers favoring university-held...copyright." On the contrary, it's easy to imagine and widely attested. Green OA does not always depend on permission from publishers. Increasingly it depends on rights retention by authors, through carefully drafted and widely supported university policies. That's a fact. My opinion is that that's a good thing. 

 You needn't share my opinion, and needn't mention the fact. But please don't misrepresent the fact or reverse my opinion.

I have read this submission. I believe that I have an appropriate level of expertise to confirm that it is of an acceptable scientific standard, however I have significant reservations, as outlined above.

# Peer review: Referee response for version 2

**DOI:** 10.5256/f1000research.9609.r14273

**Published:** 2016-06-10

**Authors:** Chris Chambers

**Affiliations:** 1 School of Psychology, Cardiff University, Cardiff, UK

All my suggestions have been addressed. I'm very happy approve this interesting and useful addition to the literature.

I have read this submission. I believe that I have an appropriate level of expertise to confirm that it is of an acceptable scientific standard.

# Peer review: Referee response for version 1

**DOI:** 10.5256/f1000research.9110.r13295

**Published:** 2016-04-28

**Authors:** Chris Chambers

**Affiliations:** 1 School of Psychology, Cardiff University, Cardiff, UK

Tennant *et al *offer a timely and insightful review of the various effects of open access publishing on science and society. The paper is well structured and enjoyable to read. Although I am not an expert on open access publishing, I also found the discussion of the literature quite balanced and evidence-based.

I have just three recommendations for revisions:

1. In the discussion of the OA citation advantage (which is excellent), the authors are very careful to avoid asserting a causal link between the OA status of a paper and the number of citations it generates. However, in my view, the conclusion of the Gargouri (2010) paper should be caveated. While the results of Gargouri are consistent with the absence of a self-selection bias in producing the OA advantage, they cannot rule it out. It might be the case that funders or institutions that mandate OA are also those that are more likely to support/host higher impact research. Furthermore, given that OA mandates are never 100% effective, perhaps authors are more likely to comply with a mandate for work they perceive to be of high impact. A useful addition to this section would be specify the conditions under which we *could* determine whether or not OA causally influences citations. This would require a randomised controlled trial in which articles are randomly are assigned to OA and non-OA routes. To my knowledge, no such a trial has yet been undertaken, although the authors will be in a more informed position to know whether this is the case.
2. There is not much discussion in the paper of the distinction between full OA and hybrid OA. Given that the APCs for hybrid OA articles tend to be substantially higher than those for full OA articles, this may warrant more prominent coverage in the economic case for OA. It is also relevant to the brief mention of the Finch Report, which (controversially) favours gold OA (including the hybrid route) over green OA.
3. I was glad to see the link formed between OA and open science more generally, as many researchers and advocates draw a distinct (and somewhat arbitrary) line between these  initiatives. In this context, it would perhaps be relevant to mention the TOP guidelines ( https://cos.io/top/). TOP is very much an evolving entity so it would be interesting to consider the inclusion of an OA standard in future revisons of TOP.

Minor

p13 This sentence is difficult to parse: "Whereas this is hyper-variable, and strongly dependent on a range of factors, it is the fact that any public interest in science that is of importance."

I have read this submission. I believe that I have an appropriate level of expertise to confirm that it is of an acceptable scientific standard.

# Peer review: Referee response for version 1

**DOI:** 10.5256/f1000research.9110.r13292

**Published:** 2016-04-25

**Authors:** Anne Tierney

**Affiliations:** 1 Department of Learning and Teaching Enhancement, Edinburgh Napier University, Edinburgh, UK

This paper is a comprehensive review of the complexities of OA. I have come late to the discussion on this paper, and I find that the previous reviewers have been meticulous in their critique of the paper, to the point I have very little to add. However, there are a couple of points for consideration. What is the effect (if any) of the UK Research Excellence Framework on Open Access? To what extent is disciplinarity a factor in Open Access? I ask this question because of the high impact of the sciences and biomedical research, but wonder about Arts and Humanities (and other areas) as a comparision. As one of the other reviewers said, "this doesn't consider the fact that the prestige of some journals is advertising in and of itself." While this is true of scientific journals, the same can't be said, for example, of education journals, so there is a lack of parity between disciplines. There was also an assumption of the willingness of reviewers to continue to offer their services freely. This aspect of OA (and subscription-based) publishing is hardly ever critiqued, but it assumed to be part of the process.

All in all, this paper gives a lot of food for thought. I don't expect a rewrite of the paper, based on my comments, but I would welcome further discussion on where the authors (and readers) see OA going in the future.

I have read this submission. I believe that I have an appropriate level of expertise to confirm that it is of an acceptable scientific standard.

# Peer review: Referee response for version 1

**DOI:** 10.5256/f1000research.9110.r13291

**Published:** 2016-04-22

**Authors:** Paige Brown Jarreau

**Affiliations:** 1 Manship School of Mass Communication, Louisiana State University, Baton Rouge, LA, USA

This is an interesting and timely review of the issue of open access to scientific literature.

The two other reviewers have highlighted specific issues that should be addressed in the revision of this article, and I agree with these issues. I've added other notes below. I think this review article would benefit from a re-write to correct potentially biased language in support of open access and to round out the review with further evidence of open access impacts on citation rates, altmetrics, scientific literacy / public engagement and research quality. 

**Notes:**

The authors cite *“fostering a culture of greater scientific literacy”* as a benefit of open access. While this is theoretically a benefit, has more or less open access in particular scientific fields been tied to greater or lesser scientific literacy in those areas? Is this potential benefit supported by research literature? The authors should be clear on what the evidence-based benefits of open access are, and also what the potential drawbacks are. References to related research should be provided on this topic.

The structure the authors use for laying out their evidence and the language they use (e.g. *“[the] case for Open Access”*) appear to lean more toward the positive impacts / benefits of open access from the outset. The authors should be very careful to review the evidence first before making value-based statements or arguments about open access, even if the evidence-based benefits outweigh any potential drawbacks, or lack of significant benefits, in the end.

Correct the typo in the following sentence: *“[In] A longitudinal study Eysenbach (2006) compared…”*

The authors write: *“One alternative explanation for the citation advantage could be that researchers choose to publish OA when a finding is more impactful, but empirical evidence contradicts this selection effect. Gargouri et al. (2010) compared citation counts within a cohort of OA articles that had either been self-selected as OA or mandated as OA (e.g., by funders). The study concluded that both were cited significantly more than non-OA articles. As such, these findings rule out a selection bias from authors as the cause for the citation advantage (Gargouri et al., 2010).” *However, couldn’t funded research also have a tendency to be considered “more impactful,” because it was chosen in the first place to be funded and mandated as OA? The authors should discuss this, and whether there is any research that experimentally investigates whether open access provides a citation advantage. This could perhaps be suggested as future research. The authors should also discuss how/why some studies have found no citation advantage for OA papers.

Related to social media mentions of research papers and citation counts, the authors might also consider citing Liang, X., Su, L. Y. F., Yeo, S. K., Scheufele, D. A., Brossard, D., Xenos, M., ... & Corley, E. A. (2014). Building Buzz (Scientists) Communicating Science in New Media Environments. *Journalism & Mass Communication Quarterly*, 1077699014550092. [ PDF]

                          

In discussing the economics of OA, the authors should also discuss any evidence of potential drawbacks for various stakeholders, such as where funds for pay-to-publish fees will come from and how these fees may affect individual researchers. Pay-to-publish models of OA may also burden early career researchers and researchers working in fields where research grants are more difficult to obtain. 

The authors do not discuss the potential impact of OA on research quality or reproducibility (reproducibility is only mentioned in the context of open data). As this has been a controversial issue in the past (e.g. the mentioned 'sting' operations) the authors should discuss any research that has investigated the impact of open access on the rigor of peer reviews, research quality, presence of replication studies / reproducibility, etc. There has also been some discussion of whether open peer review (like that used by F1000Research) affects the quality of reviews, e.g. (Rooyen *et al. *1999). The authors should mention this and/or subsequent literature when addressing open peer review.

There have also been studies on scientists' / journal article authors' perceptions and attitudes toward open access, e.g. Schroter and Tite (2005; 2006). The authors might considering summarizing some of this research, as it gives context to some of the existing barriers to open access and perceived drawbacks among researchers.

Summary:

In summary, the topic of this review is important and timely. However, this paper falls short of what I would expect from a systematic review in terms of systematically summarizing previous research findings related to the impact of open access on scientific publishing, public engagement with science, science literacy and altmetrics. The authors should be careful to hold back value judgements / arguments related to the case for open access until having systemically reviewed the evidence-based benefits, drawbacks, and/or lack of significant benefits. The authors should also provide some discussion of how we might objectively weigh any evidence-based benefits with potential drawbacks for various stakeholders including researchers and especially early career researchers. The authors should avoid summarizing mostly the findings of previous studies that find positive impacts of open access on the various domains of potential impact they consider in their paper. The evidence already presented in the paper is rigorous and detailed. However, I would recommend a revision that rounds this review out with more systematic evidence.

I have read this submission. I believe that I have an appropriate level of expertise to confirm that it is of an acceptable scientific standard, however I have significant reservations, as outlined above.

## References

[1] LiangXSuLYeoSScheufeleDBrossardDXenosMNealeyPCorleyE: Building Buzz: (Scientists) Communicating Science in New Media Environments. *Journalism & Mass Communication Quarterly*.2014;91(4) : 10.1177/1077699014550092 772-791 10.1177/1077699014550092

[2] van RooyenSGodleeFEvansSBlackNSmithR: Effect of open peer review on quality of reviews and on reviewers' recommendations: a randomised trial. *BMJ*.1999;318(7175) : 10.1136/bmj.318.7175.23 23-27 10.1136/bmj.318.7175.23 PMC276709872878

[3] SchroterSTiteLSmithR: Perceptions of open access publishing: interviews with journal authors. *BMJ*.2005;330(7494) : 10.1136/bmj.38359.695220.82 756 10.1136/bmj.38359.695220.82 15677363PMC555876

[4] SchroterSTiteL: Open access publishing and author-pays business models: a survey of authors' knowledge and perceptions. *J R Soc Med*.2006;99(3) : 10.1258/jrsm.99.3.141 141-8 10.1258/jrsm.99.3.141 16508053PMC1383760

# Peer review: Referee response for version 1

**DOI:** 10.5256/f1000research.9110.r13430

**Published:** 2016-04-18

**Authors:** Peter Suber

**Affiliations:** 1 Berkman Center for Internet & Society, Harvard University, Cambridge, MA, USA

The article is very well-done, unusually thorough and detailed. Here are a few ways to improve it.

When I refer to page numbers, I mean the page numbers in the PDF of v1, April 11, 2016.

http://f1000research.com/articles/5-632/v1

"You" refers to the authors.

Apologies in advance if I sometimes cite my own work in these comments. 

p. 1. In the abstract you say, "The economic case for Open Access is less well-understood, although it is clear that access to the research literature is key for innovative enterprises, and a range of governmental and non-governmental services." 

This understates the economic case. For example, some subscription journals convert to OA precisely for economic benefits.

See the preliminary version of David Solomon, Bo-Christer Björk, and Mikael Laakso, "Converting Scholarly Journals to Open Access: A Review of Approaches and Experiences" now open for public comment. (The final version will be published this summer.)

https://osc.hul.harvard.edu/programs/journal-flipping/public-consultation/

See especially section 4.6, "Increased revenue and financial viability."

https://osc.hul.harvard.edu/programs/journal-flipping/public-consultation/4/6/

p. 3. You say, "The Green route refers to author self-archiving, in which a version of the peer-reviewed article is posted online to a repository or website."

Green OA also applies to preprints, which are not peer-reviewed.

p. 3. You say, "This [green] route is dependent on journal or publisher policies on self-archiving ( sherpa.ac.uk/romeo)."

This is importantly incomplete. It overlooks rights retention. Some individual authors retain enough rights to authorize green OA on their own. While this may be fairly rare, rights-retention OA policies at universities are increasingly common. More than 80 institutions in North America, Europe, Africa, and Asia have now adopted rights-retention OA policies. Under these policies, the faculty grant non-exclusive rights to their institution before they sign future publishing contracts. The institution then has permission to make those future articles OA without having to seek permission from publishers. (The institutions also grant the same non-exclusive rights back to authors.)

For more detail on rights-retention OA policies, see Stuart Shieber and Peter Suber, "Good Practices for University OA Policies." 

http://bit.ly/goodoa 

p. 3. You say, "A subscription to all peer-reviewed journals is not affordable for any single individual, research institute or university (Odlyzko, 2006)." 

This is true and important, but it's a pity you don't cite more recent evidence than 2006. 

An important kind of evidence for this proposition is that not even Harvard University can afford all the journals needed by its faculty and students, and must cancel journals every year for budgetary reasons alone. I've collected seven public statements from Harvard to this effect (2008-2012) in the supplements to p. 30 of my 2012 book (Open Access, MIT Press, 2012).

http://bit.ly/oa-book#p30.2

p. 3. You say, "Much of the driving force behind this global change has been through a combination of direct, grassroots advocacy initiatives in conjunction with policy changes from funders and governments."

Please add *university policies* to this list. They're on a par with funder policies in importance, and they're far more numerous. ROARMAP shows that 7+ times more universities have OA policies than funders. 

p. 3. You say, "The Open Access movement is intrinsically tied to the development of the Internet and how it redefined communication and publishing (Laakso et al., 2011)."

For more documentation on how the OA movement arose as soon as the internet arose, see my Timeline of the Open Access Movement.

http://legacy.earlham.edu/~peters/fos/timeline.htm

In 2009, I moved the timeline to the Open Access Directory wiki, and you should probably cite that version:

Timeline of the open access movement...

http://oad.simmons.edu/oadwiki/Timeline

...especially subsection on developments before 2000.

http://oad.simmons.edu/oadwiki/Timeline_before_2000

(You might cite this timeline again on p. 5, when you introduce your own timeline.)

p. 3. You say, "One result of the growing OA movement is the rise of OA-only publishers...."

Somewhere in this paragraph, I'd mention that some OA publishers are for-profit (e.g. BMC) and some are non-profit (e.g. PLoS).

p. 4. Your section on the impact advantage is very well-done. Most treatments are much briefer, less careful, and less detailed than yours.

I have just these suggestions. You cite authors of individual studies, and Alma Swan's 2010 literature review. But you don't cite the mother lode of literature on this topic: Steve Hitchcock's annotated bibliography, "The effect of open access and downloads ('hits') on citation impact: a bibliography of studies."

http://eprints.soton.ac.uk/354006/1/oacitation-biblio-snapshot0613.html

Or more precisely, you cite it once, 10 paragraphs before the section on the impact advantage begins. You should cite it again within the section on the impact advantage. You should mention that it's comprehensive and annotated.

Hitchcock stopped updating it in 2013. But you should mention that SPARC Europe has committed to update it through its Open Access Citation Advantage Service.

http://sparceurope.org/oaca/

Finally, in the same place where you cite Swan's literature review, you should cite Ben Wagner's literature review, "Open Access Citation Advantage: An Annotated Bibliography," Issues in Science and Technology Librarianship, Winter 2010.

http://www.istl.org/10-winter/article2.html

p. 5. In the timeline entry for 2002, the BOAI was released on February 14, not January 14.

p. 6. In the timeline entry for 2013, I'd say that the suicide of Aaron Swartz "increases" (not "gains") international attention for the OA movement, or "draws new attention" to the OA movement. The current language suggests that the OA movement didn't have international attention before that, which is very far from the truth.

p. 9. You say, "Shifting copyright to stay with the author allows for wider re-use, including TDM, and forms the basis for a robust and developing public domain." 

You shouldn't use "public domain" here. In copyright law, the term has a specific meaning which you don't mean here.

p. 10. You say, "Only recently has any transparency into the detailed costs of subscriptions been gained by using Freedom of Information Requests to bypass non-disclosure agreements between libraries and publishers (Lawson & Meghreblian, 2015)."

Here you overlook the earlier Big Deal Contract Project in the US, from Ted Bergstrom, Paul Courant, and Preston McAfee. It too used public records laws and Freedom of Information requests. I'm not sure when it launched, but it was before 2009.

http://www.econ.ucsb.edu/~tedb/Journals/BundleContracts.html

p. 10. You say, "The average production cost for one paper is estimated to be around $3500–$4000 (Van Noorden, 2013)."

I've seen dozens of widely varying estimates of this cost, most of them much lower than Van Noorden's. Unfortunately I don't have time to hunt them down. I hope you can introduce at least a few more, if only to show that estimates differ widely here.

p. 10. You say, "Philip Campbell (Editor-in-Chief of Nature) stated that his journal’s internal costs were at $20,000–$30,000 per paper...."

To clarify, I think he meant that this was the cost per **published **paper. If Nature rejects x articles for every one it publishes, then this includes the cost of peer reviewing x rejected articles. Since Nature is very selective, x is high. But this "cost per published paper" should not be compared to costs for peer-reviewing a single paper or the production costs of publishing an accepted paper.

p. 10. You say, "OA publishing is most prevalent in the form of ‘pay-to-publish’...."

This is either false or misleading. About 70% of peer-reviewed OA journals charge no APCs at all. In that sense, the fee-based model is not the most prevalent. It's a minority model. On the other hand, about 50% of the articles published in peer-reviewed OA journals are published in the fee-based variety. 

On my claim that most OA journals charge no APCs: 

See my article, "Good facts, bad predictions," SPARC Open Access Newsletter, June 2006.

https://dash.harvard.edu/handle/1/4391309

And my article, "No-fee open-access journals," SPARC Open Access Newsletter, November 2, 2006. https://dash.harvard.edu/handle/1/4552050

The DOAJ used to make it easy to see what percentage of listed journals were fee-based and what percentage were no-fee. But it has temporarily made that difficult by combining the categories of "no-fee journals" and "journals for which we don't have enough information to say."

On my claim that about half the articles published in peer-reviewed OA journals are published in the fee-based variety, see the updates to p. 170 of my 2012 book. There I cite three studies and quote the relevant excerpts.

http://bit.ly/oa-book#p170

p. 10. When you describe ways in which fee-based OA journals mitigate some problems arising from the model, you mention the firewall between the editorial and business side of the journal (good), and you mention fee waivers (good).

You should also mention fee discounts, which many journals give in lieu of fee waivers. 

You should also mention that most fees charged by fee-based journals are paid by funders (59%), or the author's employer (24%), and only 12% are paid by authors themselves. These numbers are from Suenje Dallmeier-Tiessen et al., "Highlights from the SOAP project survey. What Scientists Think about Open Access Publishing," arXiv, January 29, 2011, Table 4.

http://arxiv.org/abs/1101.5260

p. 12. You say, "In his article Sutton (2011)..."

please change "his" to "her". The article is by Caroline Sutton.

p. 12. You say, "While OA is not a solution to all aspects of research accessibility (e.g., language barriers and disability access remain continuing issues to be addressed)...."

See my 2012 book (Open Access, MIT Press, 2012, http://bit.ly/oa-book), at pp. 26-27, where I make much the same point. "OA isn't universal access" and by itself doesn't overcome "filtering and censorship barriers", "language barriers", "handicap access barriers", or "connectivity barriers".

p. 13. You refer to "the fact that access to knowledge is actively prohibited in fields like public health...."

I don't know what you mean here by "actively prohibited".

p. 13. You say, "Some traditional publishers, and some academics, have argued that public access to research is not required because research papers cannot be understood by non-specialists...."

Here you might want to cite Section 5.5.1 ("OA for Lay Readers," pp. 115-119) of my 2012 book.

p. 13. You say, "The shift from a ‘reader pays’ to an ‘author pays’ mode...."

I recommend avoiding the term "author pays" for the reasons I gave in my fourth comment to p. 10 above. Most OA journals don't charge author-side fees, and among those who do, most fees are not paid by authors. 

p. 13. You say, "This has been at least partially mitigated with waiver fees for authors from developing countries and additional provisions in research grants...."

Yes. But again, don't forget that the majority of peer-reviewed journals are no-fee journals. See my fourth comment to p. 10 above. 

pp. 15-15. You say, "Fortunately, it seems that funders and research organisations are moving in that direction. Since 2005, the number policies supporting OA publishing increased steadily. Consequently, it is now the responsibility of researchers to ensure OA to their publications either by choosing the green or the gold road."

Since you're recapitulating some grounds for optimism here, I'd also reiterate the growth of rights-retention OA policies. See my second comment on p. 3 above. 

p. 16. You say, "As Peter Suber, a leading voice in the OA movement, stated: 'As long as they do not have the power to stop Open Access, the toll-access publishers are not the enemy'."

Thanks for quoting me. I wanted to give you the source to cite. Unfortunately, I don't think you're using an exact quote. Here's the closest one I can find:

"TA [toll-access] publishers are not the enemy. They are only unpersuaded. Even when they are opposed, and not merely unpersuaded, they are only enemies if they have the power to stop OA. No publisher has this power, or at least not by virtue of publishing under a TA business model. If we have enemies, they are those who can obstruct progress to OA. The only people who fit this description are friends of OA who are distracted from providing OA by other work or other priorities."

It's from "Two distractions," SPARC Open Access Newsletter, May 3, 2004.

https://dash.harvard.edu/handle/1/4391169

I have read this submission. I believe that I have an appropriate level of expertise to confirm that it is of an acceptable scientific standard.

# Response

**Published:** 2016-05-10

**Authors:** Jon Tennant

**Affiliations:** Imperial College London, UK

Hi Alexander,

I've been looking into this, and the most up-to-date statistics for this based on the DOAJ come from this source: http://citesandinsights.info/civ16i4.pdf. Figures here seem to suggest that 71% of journals in the DOAJ do not levy an APC. This is likely to change slightly with the updated 'crackdown' from the DOAJ ( http://www.nature.com/news/open-access-index-delists-thousands-of-journals-1.19871), but I'll add a reference to this in.

Jon

# Response

**Published:** 2016-05-24

**Authors:** Peter Suber

**Affiliations:** Harvard University, USA

Here are the latest stats from the DOAJ (May 24, 2016).

https://goo.gl/LejTAw

# Response

**Published:** 2016-05-25

**Authors:** Jon Tennant

**Affiliations:** Imperial College London, UK

Dear Alexander,

 I'm just posting the link that Peter was kind enough to send us yesterday, with updated statistics on this matter: https://plus.google.com/+PeterSuber/posts/HjrRDcrZS8p

 Important points:

 Here are the numbers as of May 24, 2016:

 Total number of journals listed in DOAJ = 8,858

 Yes (fee-based) = 1,424 = 16%

 No (no-fee) = 2,601 = 29%

 No info = 4,833 = 55%

 Further references and information are provided in that post, and will be integrated into the next version of this manuscript.

 Best,

 Jon

# Peer review: Referee response for version 1

**DOI:** 10.5256/f1000research.9110.r13293

**Published:** 2016-04-15

**Authors:** Gwilym Lockwood

**Affiliations:** 1 Neurobiology of Language Department, Max Planck Institute for Psycholinguistics, Nijmegen, Netherlands

This is the first time I've written an open peer review, although I always sign private peer reviews. Normally I'd make comments directed to the authors and the authors alone, but since this is open, I've also included a section for other readers of this paper. This may sound a bit like an Amazon or Airbnb review or something.

Short summary for readers

This is an excellent paper about the academic, economic, and societal benefits and impacts of Open Access. It's a good introductory text for people who don't know much about OA and would like to know more. It's also a good persuasive text for stakeholders in policy, universities, publishing, funding, etc. positions who may be interested in including OA in their decision making.

In addition to its attention to detail, its main strengths are its focus, its brevity, and its relative impartiality.

One of the difficulties with writing about OA is that there are so many overlapping issues; this paper is very good at giving a brief overview or description of the other issues, pointing the reader in the direction of somewhere with more information, and then getting back onto the topic.

Another thing about OA is that its advocates are very passionate about it. As with any cause, that's a good thing for its supporters, but overwhelmingly pro-OA resources can seem potentially off-putting to neutrals. This paper does an excellent job of presenting an evidence-based pro-OA viewpoint in a measured tone and without coming across as ideological.

One possible caveat is that the paper presents extensive evidence of what OA does, but it doesn't tackle the meatier issue of how to implement it successfully. However, I feel that's a separate issue which is beyond the scope and purpose of this paper.

Suggestions and comments for authors

First of all, great article! Well done and thank you for pulling together what is a disparate collection of links and literature into a one-stop shop which is both useful and coherent. I like this article a lot... but my role here is to criticise and make it better, so the rest of this review will focus on that.

This article is well-written and well-structured. That's made it much easier as a reviewer to simply go through the article and highlight my issues with it paragraph by paragraph, rather than having to make it coherent first and then sort out the smaller things.

The vast majority of the issues I have with this paper are minor ones, so it didn't make sense to have separate major/minor sections; rather, I'll just go through them in order in the text.

(I printed this out to underline/comment on, so for me, tables 1 and 2 came during the academic case for OA section. Online, they're supplementary materials, and I think it's best that way, but this is why I'm commenting on the tables during that section)

**Abstract**

"We recommend that OA supporters focus their efforts on working to establish viable new models and systems of scholarly communication, rather than trying to undermine the existing ones..."

In general, I agree with this sentiment. However, I feel that its inclusion in the abstract is a bit jarring as the text of the article doesn't really cover recommendations to OA supporters at all, other than in the very last paragraph. I think that's good, as I feel this paper is best suited as relatively neutral source of information rather than a preaching to the converted or ideology discussion kind of purpose. So, I think this part can be left out of the abstract; it doesn't refer to any particular "recommendations to OA supporters" bit in the text and potentially clouds the strength of the relative impartiality of the paper.

**A brief history of OA**

"BioMed Central and ... PLOS were founded in the early 2000s and remain successful businesses to date." (p3, col2)

Technically, PLOS is a non-profit. I suggest changing *successful businesses* to successful business models. This both highlights the financial sustainability of OA (increased APCs at PLOS notwithstanding) and also sets it apart from traditional publishers, which are definitely successful businesses.

**The academic case for OA**

figure 1 (p4, top)

I have difficulty interpreting the y-axis on figure 1. It's labelled as cumulative number of PubMed articles relative to 2000, but I'm not sure how to read it. Reading off 2014, non-OA is c.22 on the y-axis, and OA is c.33 on the y-axis. Based on the figure 1 caption about the ratio, I'm interpreting this as meaning that, in 2014, the ratio of cumulative PubMed articles was approx 33:22 OA to non-OA, or in other words, 60% of PubMed articles in 2000-2014 were OA. However, I'm not sure if this is how it's meant to be interpreted. I think that it's well visualised, and really makes it clear how OA has taken off, but exactly what the numbers represent on the y-axis is unclear to me: number of articles? number of times more articles? It could use some relabelling.

"Napster moment" (p4, col1)

I like the comparison, but it could use a citation (even just the Napster wikipedia article) and/or a little more explanation to clarify what that means.

Table 1:

"1991 ... by the American physician Paul Ginsparg"

He's a physicist, not a physician.

Table 2:

Xu et al (2011)

I don't think this reference was very well cited. Firstly, *Oxford Open Journals* are listed as a discipline, when they're the source of papers across disciplines. The actual disciplines were Medicine, Social Sciences, Mathematics & Physical Sciences, Life Sciences, and Humanities. Secondly, you list the citation advantage as 138.87%. However, one of the main findings of this paper was the disparity in citation advantages; it ranged from 163.16% for OA articles in Mathematics & Physical Sciences to an actual citation disadvantage of -49.24% for OA articles in Humanities. Given the pro-OA nature of the paper, I feel like you have an extra responsibility to report the few anti-OA pieces of evidence.

Gargouri et al (2010) (page 8, col1)

This paragraph is about a possible confound for the OA citation advantage, where it could be that researchers choose to publish OA for extra cool findings, and you use the Gargouri et al. study to counter this... which is totally correct. You write:

"Gargouri et al. (2010) compared citation counts [for articles which were] self-selected as OA or mandated as OA. The study concluded that both were cited significantly more than non-OA articles. As such, these findings rule out a selection bias"

This is true that both OA types were cited more than non-OA. However, it's also missing the crucial point that there was no difference in citation between self-selected OA articles and mandatory OA articles. Including this would strengthen your point to show that it's OA itself which leads to the citation advantage.

The whole section about altmetrics (subhead societal impact of the academic case for OA, p8, col2) could use some attention. It's not clear until much later what the difference is between alternative metrics (i.e. altmetrics), i.e. the various types of metrics which are alternative to journal impact factors, and Altmetric, i.e. the company which is often confusingly referred to as Altmetrics (not in this paper, to be fair, but elsewhere). A quick disambiguating sentence or two would be really useful here.

In the following paragraph (page 8, col2), you write about OA altmetrics advantage, and say that there's a logical assumption that OA articles should have one. However, this doesn't consider the fact that the prestige of some journals is advertising in and of itself. You can, and do, get a lot of closed-access papers which generate high altmetrics (social media attention, Mendeley readhership) from academics who do have access. And sure enough, in the next paragraph, (page 8 and 9), the Wang et al. 2015 article finds that the OA altmetric advantage doesn't extend to the most impactful articles. I think this section can be made more nuanced and informative by quickly discussing the role of journal prestige. Nothing in depth, just as something that exists and needs to change (for example, you could point people to Brembs *et al. *and the Deep Impact paper in Frontiers).

"Essentially, copyright is a tool wielded by traditional publishers for financial gain rather than fostering creativity..."

I don't disagree with this. However, I feel it comes on too strong. I think it's fair to say that most people's immediate opinion of copyright is "well, I'd like my stuff to be copyrighted, as that means people can't steal it and pass it off as their own". I think that you need a little more detail here, even just two or three sentences to explain how and why copyright is used for financial gain rather than author protection. Otherwise, it just sounds political/ideological, and counterintuitive for people who haven't read much about copyright.

Glenisson et al. (2005) citation (page 9, col2).

You write that TDM has "proven to be useful for a large variety of applications", and use the Glenisson citation to back this up. I have to say here that I know very little about TDM; however, following through to the Glenisson paper, I don't see how it supports that conclusion. I read it and it seems to show a proof-of-concept kind of study: that TDM can group a set of papers into themes in the same way that an expert can. This is really cool and everything, but I don't think that that substantiates your point that TDM is useful for a large variety of applications. Rather, I'd like to see a couple of specific examples, which you then describe more fully in the next paragraph. One good one is Swanson 1987 (I think - taken from here: http://people.ischool.berkeley.edu/~hearst/papers/acl99/acl99-tdm.html), who used TDM to make the link between migraines and magnesium deficiency.

"...simply because one can no longer keep up with the published literature".

Small point, but I think it's worth stressing that this is due to the amount of literature that there is.

**The economic case for OA**

the pay-to-publish part (p10, col2)

I feel this glosses over problems with pay-to-publish. You come back to predatory OA later, but this isn't quite the same: I think it could use a couple of extra sentences describing what the conflict of interest for researchers is, and also stress that pay-to-publish makes it potentially in a journal's interest to accept more papers than they necessarily should. One of the most common anti-OA arguments I see in non-scientific media is that OA is pay-to-publish, which is often misrepresented as "pay-to-publish is publication bribery". I think this section needs a little more substance to it to acknowledge/address this.

"making publication costs dependent on the value added..." (page 11, col1)

When talking about the value added by journals, this paragraph ignores the elephant in the room: journal prestige. Again, I know that this isn't the purpose of this article, but I think it could really be strengthened by mentioning it before moving on.

"Much primary research actually takes place outside of academia inside research and development departments" (page 11, col2)

The part following this sentence is muddy. First, you talk about R&D outside academia (i.e. presumably private research), and then you talk about access to research results because they're publicly financed public goods. So, what does that mean, that R&D from private businesses who've invested their own capital in it should be made available to all? (maybe I agree with that, in some cases, but a lot of people sure won't)

I think this paragraph could be honed a bit; otherwise, it's straying into the ideological territory of saying that all private research should be made public for the public good. That transcends OA in scholarly publishing, and makes OA in scholarly publishing too easy to dismiss.

The cancer research paragraph (page 12, col1) is also unclear. It took me a while to figure out it's talking about UK expenditure - my first assumption of "total expenditure" meant worldwide. It's also not totally clear what the point is - the geographical origin of research is unrelated to its open status. I think that it's quite a leap to write (apologies for paraphrasing) "83% of UK economic benefit from cancer research comes from research outside UK, therefore open access is good", because I think it conflates two different things.

Also, small point, "17% of the annual net-monetary was estimated" is missing the word benefit after net-monetary.

**The societal case for OA**

Small point: as somebody who wears a linguistics hat quite often, it rankles to read on page 11 "Examples of [non-academic] groups who might benefit include... those who work in linguistics and translation". Translation, for sure, but linguistics is an academic field - you even mention the Lingua to Glossa movement organised by academic linguists later in the manuscript! To me, this is like writing "...those who work in biology and vets", lumping the academic field and a practical use of that field together. Just referring to translation is fine.

Citizen engagement (page 13, col 1)

I agree that these are great examples of citizen engagement with science, but at the risk of sounding like an Elsevier representative, interest in projects like Galaxy Zoo does not entail desire to download and read papers. In fact, you could even make the (spurious) argument that those projects come into existence precisely because citizens aren't interested in downloading and reading papers. I don't actually agree with that, I agree with your general point... but I think that citizen science project interest and citizen science paper interest. Obviously I think it is in the public interest to have science journals OA, but this isn't the right argument (and I think the sentence "Such statements conflate a lack of desire or need for access with the denial of opportunity to access research" is perfect). I think a stronger argument would be to look at existing OA journals, such as PLOS and Frontiers, and see how many views and downloads come from people who aren't academics. If you can point to, say,  some of the most viewed/downloaded PLOS papers and say "look, 30% (or whatever, that's a random number) of these readers aren't academics, they're real people who are interested in it", that would make for a stronger argument.

Quibble about the "yes, we were warned about Ebola" example: the finding from that paper (that Liberians have Ebola antibodies in their blood, suggesting the endemic presence of Ebola) is actually written on the first page preview of the paper ( http://www.sciencedirect.com/science/article/pii/S0769261782800282/part/first-page-pdf, accessed from my laptop outside my institution). It could be argued that anybody could see this finding anywhere in the world, meaning that it's not a problem of OA, it's a problem about searching and indexing. A good counterargument to *that *is obviously that this paper would have been unsearchable with TDM at the start of the outbreak when people were combing through all West African Ebola literature.

" 'green' model of OA adoption" (page 13, col2)

You generally refer to Green and Gold routes, with the colours capitalised. Just a small terminology thing to keep consistent.

A much more important thing is also on page 13, col2:

"The pay-to-publish system is a potentially greater burden for authors in **developed **countries, considering that they are not used to paying publication costs, and funding systems for OA are not as well-established as those in the Western world."

--> developing countries, not developed countries!

Predatory publishers (page 14, col1)

I agree with Ross Mounce's comment on the paper: you give Beall too much importance. I think it can be a useful list and should be mentioned, but definitely include some caveats like the ones Ross writes, or the fact that he added Frontiers to the list because of a couple of editorial mistakes.

**Conclusions**

Peter Suber (page 16, col1)

You describe him as "a leading voice in the OA movement", but I think you should write what his positions are (see http://cyber.law.harvard.edu/~psuber/wiki/Peter_Suber) in order to justify his importance.

**Other general things**

There are no proposed solutions in this paper, which is totally fine, because it's beyond the scope of the paper. I feel it could benefit by putting in a couple of sentences here and there about who is needed for driving this change: academics, funders, governments, etc.

I was disappointed not to see anything about the Dutch government and university library organisations' collective drive towards OA. They've changed the national law on copyright, they've reached agreements with most major publishing groups, they may well introduce mandatory OA publishing in the Netherlands in 2016, and they've made it one of the main priorities of their EU presidency this year. It's like the best example of how a whole country can take the lead and sort it out. I think including a quick reference to the Netherlands as an example of excellent OA policy (in the same way that you mention sciELO in Latin America) would go a long way towards convincing the people who are reading this thinking, "ah, yes, I guess OA makes sense in the developing world, but we're doing fine here in the West and it would be too difficult to change things". A good summary of that is here: http://openaccess.nl/en/in-the-netherlands/current-situation

Final remarks

That's the end of my 2800-odd word review. I really enjoyed reading this paper, going through it, and trying to find ways to improve it. Thanks to the authors for writing an excellent paper.

I have read this submission. I believe that I have an appropriate level of expertise to confirm that it is of an acceptable scientific standard.

## References

[1] BrembsBButtonKMunafòM: Deep impact: unintended consequences of journal rank. *Front Hum Neurosci*.2013;7: 10.3389/fnhum.2013.00291 291 10.3389/fnhum.2013.00291 23805088PMC3690355

[2] SwansonD: Two medical literatures that are logically but not bibliographically connected. *Journal of the American Society for Information Science*.1987;38(4) : 10.1002/(SICI)1097-4571(198707)38:4<228::AID-ASI2>3.0.CO;2-G 228-233 10.1002/(SICI)1097-4571(198707)38:4<228::AID-ASI2>3.0.CO;2-G
